# Genetic and epigenetic features of promoters with ubiquitous chromatin accessibility support ubiquitous transcription of cell-essential genes

**DOI:** 10.1093/nar/gkab345

**Published:** 2021-05-12

**Authors:** Kaili Fan, Jill E Moore, Xiao-ou Zhang, Zhiping Weng

**Affiliations:** Program in Bioinformatics and Integrative Biology, UMass Medical School, Worcester, MA, USA; Program in Bioinformatics and Integrative Biology, UMass Medical School, Worcester, MA, USA; Program in Bioinformatics and Integrative Biology, UMass Medical School, Worcester, MA, USA; Program in Bioinformatics and Integrative Biology, UMass Medical School, Worcester, MA, USA

## Abstract

Gene expression is controlled by regulatory elements within accessible chromatin. Although most regulatory elements are cell type-specific, a subset is accessible in nearly all the 517 human and 94 mouse cell and tissue types assayed by the ENCODE consortium. We systematically analyzed 9000 human and 8000 mouse ubiquitously-accessible candidate cis-regulatory elements (cCREs) with promoter-like signatures (PLSs) from ENCODE, which we denote ubi-PLSs. These are more CpG-rich than non-ubi-PLSs and correspond to genes with ubiquitously high transcription, including a majority of cell-essential genes. ubi-PLSs are enriched with motifs of ubiquitously-expressed transcription factors and preferentially bound by transcriptional cofactors regulating ubiquitously-expressed genes. They are highly conserved between human and mouse at the synteny level but exhibit frequent turnover of motif sites; accordingly, ubi-PLSs show increased variation at their centers compared with flanking regions among the ∼186 thousand human genomes sequenced by the TOPMed project. Finally, ubi-PLSs are enriched in genes implicated in Mendelian diseases, especially diseases broadly impacting most cell types, such as deficiencies in mitochondrial functions. Thus, a set of roughly 9000 mammalian promoters are actively maintained in an accessible state across cell types by a distinct set of transcription factors and cofactors to ensure the transcriptional programs of cell-essential genes.

## INTRODUCTION

Cells in a multicellular organism share the same genome but interpret it differently to carry out cell type-specific transcriptional programs. Cell-type specificity is partly manifested in maps of chromatin accessibility (1,2), DNA methylation (3), and histone modifications (4) and partly in the levels of regulatory proteins such as transcription factors (5). Among the three types of maps, chromatin accessibility is a powerful indicator of whether a genomic region may have regulatory functions, while DNA methylation and histone modifications suggest the type of regulatory functions (e.g. promoters, enhancers or insulators). DNase-seq (6) and ATAC-seq (7) are two widely used techniques for mapping chromatin accessibility, and they have revealed that chromatin accessibility maps are highly variable across cell and tissue types (8–10).

In an early study, we combined the DNase assay with microarrays to map chromatin accessibility in 1% of the human genome in six cell lines; we observed that 22% of the DNase hypersensitive sites (DHSs) were shared among all six cell lines (11). We called these sites ubiquitous DHSs and found them to be enriched in promoters and insulators, while the cell type-specific DHSs were enriched for enhancers (11). It was unclear whether this set of ubiquitous DHSs would remain a distinct group as chromatin accessibility maps became available for a larger number of biosamples, and if so, whether we could discern more biological features for the group beyond just the enrichment in promoters and insulators.

As part of the ENCODE Project, we recently identified a set of 2.2 million representative DNase hypersensitive sites (rDHSs) by integrating ∼70 million DHSs identified in >500 DNase-seq experiments across diverse human cell and tissue types (12). We further defined a subset of rDHSs with high ChIP-seq signals (defined as *Z*-score > 1.64) of two key histone modifications (H3K4me3 and H3K27ac) or the chromatin-structure protein CTCF as candidate *cis*-regulatory elements (cCREs) (12). cCREs were further classified according to whether they scored high signals in the four assays (DNase-seq and ChIP-seq of H3K4me3, H3K27ac and CTCF) and also based on their distances from GENCODE-annotated transcription start sites (TSSs), including a group of cCREs with promoter-like signatures (PLSs), another group of cCREs with enhancer-like signatures (ELSs; further divided into TSS-proximal pELS and TSS-distal dELS using a distance cutoff of 2 kb), and three other groups (12). When we examined the cell type specificity of cCREs in the 25 human biosamples with data from all four assays, we observed that cCRE-PLSs tended to be active (defined as having high DNase or ChIP-seq signals) in multiple biosamples while cCRE-ELSs tended to be active in only a few biosamples (12), consistent with earlier observations of promoter and enhancer activity (8,11,13).

In this study, we investigated whether our previous observations about ubiquitous DHSs (11) held using the ENCODE Registry of cCREs and the extensive collection of ENCODE and Roadmap Epigenomics data. We defined 15 989 ubiquitous human rDHSs and 13 247 ubiquitous mouse rDHSs (ubi-rDHSs) as those rDHSs with high DNase signals in at least 95% of DNase-seq experiments. Confirming our previous results (11), we found that nearly 60% of ubi-rDHSs had promoter-like signatures (referred to as ubi-PLSs), and ∼20% additional ubi-rDHSs were TSS-proximal enhancers (within 2 kb of a GENCODE-annotated TSS). In particular, ubi-PLSs are highly enriched in the promoters of cell-essential protein-coding genes. We found that ubi-PLSs are a set of regulatory elements with distinct properties. Compared with the remaining cCRE-PLSs (called non-ubi-PLSs), ubi-PLSs are highly enriched in CG dinucleotides and are depleted in the TATA-box. Additionally, ubi-PLSs are enriched in the motifs of ubiquitously expressed transcription factors and preferentially bound by transcriptional cofactors that regulate ubiquitously expressed genes; thus, the binding of the transcriptional machinery and regulatory proteins is the driving force behind the ubiquitous chromatin accessibility of ubi-PLSs. Furthermore, ubi-PLSs are highly conserved between human and mouse at the synteny level and show a unique pattern of nucleotide diversity in human populations—high at the ubi-PLS and low at flanking regions. ubi-PLSs are more enriched than non-ubi-PLSs in genes implicated in Mendelian diseases that impact most cell types, in particular, deficiencies in mitochondrial functions. In conclusion, there is a distinct set of ∼9000 promoters in mammalian genomes actively maintained in the open chromatin state in nearly all cell types by a distinct set of transcription factors and cofactors to ensure the transcriptional program of cell-essential genes.

## MATERIALS AND METHODS

### Definition of ubi-rDHSs and ubi-PLSs (Figures 1A–C and 6A–B, Supplementary Figure S1A–B and Supplementary Table S2)

**Figure 1.**
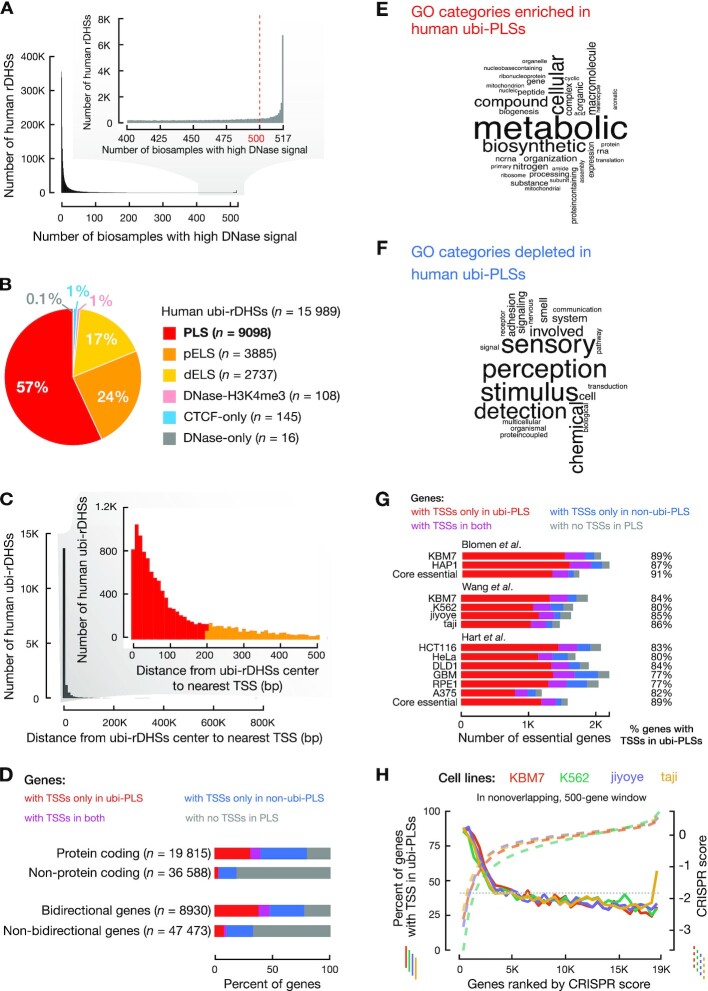
The majority of ubiquitously DNase-accessible regions are active promoters of essential genes. (**A**) Definition of ubi-rDHSs. The histogram shows the number of human rDHSs that have high DNase signals in different numbers of biosamples. rDHSs that have high DNase signals in 500 or more human biosamples (out of a total of 517 biosamples) are defined as ubi-rDHSs. (**B**) The majority of human ubi-rDHSs have promoter-like signatures or are TSS-proximal with enhancer-like signatures. The pie chart shows the category of ubi-rDHSs: PLS, rDHSs with promoter-like signature; pELS, TSS-proximal enhancer-like signature; dELS, TSS-distal enhancer-like signature; DNase-H3K4me3, TSS-distal rDHSs with high DNase and high H3K4me3 signals but low H3K27ac signals; CTCF-only, rDHSs with high DNase and high CTCF signals but low H3K4me3 and H3K27ac signals; DNase-only, rDHSs with high DNase signals but low H3K4me3, H3K27ac and CTCF signals. (**C**) The majority of ubi-rDHSs are near a TSS. The histogram shows the number of ubi-rDHSs at a certain distance from the nearest GENCODE-annotated TSS. (**D**) Higher percentages of protein-coding genes and bidirectional genes have TSSs overlapping ubi-PLSs than other types of genes. Genes with TSSs only overlapping ubi-PLSs are shown in red; genes with TSSs only overlapping non-ubi-PLSs are in blue; genes with TSSs overlapping both ubi-PLSs and non-ubi-PLSs are in purple; and genes with no TSSs overlapping cCRE-PLSs are in gray. (**E**) Word cloud of the most enriched GO Biological Process terms for genes with TSSs overlapping ubi-PLSs. (**F**) Word cloud of the most depleted GO Biological Process terms for genes with TSSs overlapping ubi-PLSs. (**G**) A high percentage of cell-essential genes have TSSs overlapping ubi-PLSs. Three groups of bar plots represent cell-essential genes from three different studies, while core essential genes overlap essential genes between different cell lines defined in the studies. Classification of genes in this figure are the same as in Figure 1D. (**H**) The TSSs of most top-ranked cell-essential genes are located in ubi-PLSs. Four solid lines show the percentage of cell-essential genes with TSSs overlapping ubi-PLSs, plotted for nonoverlapping, 500-gene windows in four cell lines (y-axis on the left, solid lines) as functions of the ranks of the genes according to their CRISPR scores. Cell-essential genes from Wang *et al.* CRISPR scores (*y*-axis on the right, dashed lines) are also shown as functions of the ranks of these scores. The horizontal dotted line shows the percentage of all tested genes with TSSs overlapping ubi-PLSs.

To define ubi-rDHSs, we started with rDHSs defined by the ENCODE consortium (12) (Supplementary Table S1A and S1B) and calculated the DNase signals (expressed as a *Z*-score) for each rDHS in all biosamples (517 in total for human and 94 in total for mouse). Following the method by the ENCODE consortium (12), a high signal is defined as *Z*-score >1.64, with the threshold of 1.64 corresponding to a *P*-value of 0.05 in a one-sided *Z*-test. We defined ubi-rDHSs as having high DNase signals in 500 or more human biosamples or 90 or more mouse biosamples. We arrived at 15 989 human ubi-rDHSs (from 2 157 387 human rDHSs; Figure 1A and Supplementary Table S2A) and 13 247 mouse ubi-rDHSs (from 1 192 301 mouse rDHSs; Figures 6A and Supplementary Table S2D). The remaining rDHSs were called non-ubi-rDHSs. We assigned cCRE-PLSs to genes or TSSs using GENCODE v24 (human) and GENCODE vM18 (mouse). Genes with TSSs overlapping ubi-PLSs are listed in Supplementary Table S2B (human) and Supplementary Table S2E (mouse). TSSs overlapping ubi-PLSs are listed in Supplementary Table S2C (human) and Supplementary Table S2F (mouse).

The ENCODE consortium defined rDHSs with high ChIP-seq signals of the H3K4me3 or H3K27ac histone modifications or the CTCF transcription factor as cCREs (Supplementary Table S1A and S1B) (12). The cCREs were further classified into groups: having promoter-like signatures (PLSs), having enhancer-like signatures (ELSs; further classified as proximal or distal to an annotated TSS), with high H3K4me3 signals but are distal to annotated TSSs (DNase-H3K4me3), or bound by CTCF but do not have high H3K4me3 or H3K27ac signals (CTCF-only) (12). We examined ubi-rDHSs by these cCRE categories (Figures 1B, 6B), as well as their distance distribution to the nearest TSS (Figure 1C).

We plotted the length distribution of all ubi-PLSs and all non-ubi-PLSs (Supplementary Figure S1A), as well as the length distribution of a randomly selected subset non-ubi-PLSs that matched the length distribution of ubi-PLSs (Supplementary Figure S1B).

### Enrichment analysis of genes associated with ubi-PLSs (Figure 1D, G, H and Supplementary Figure S1C–F)

We examined the types of genes using the GENCODE v24 basic annotation. We discarded the GENCODE TSSs of transcripts with inactive or uncertain biotypes such as pseudogenes and TEC (to be experimentally confirmed). In total, there are 19 815 and 36 550 GENCODE genes with the protein-coding and other biotypes, respectively. We counted the percentages of genes in each biotype whose TSSs were located only in ubi-PLSs, only in non-ubi-PLSs, in both ubi-PLSs and non-ubi-PLSs (i.e., some TSSs of the gene whose TSSs overlapped ubi-PLSs while some other TSSs of the same gene whose TSSs overlapped non-ubi-PLSs) or not in ubi-PLSs (Figure 1D, colored accordingly).

Bidirectional genes (i.e. genes whose TSSs were within 1000 nt of each other and on opposite genomic strands) were defined based on a previous study (14). We identified 4755 pairs formed by 8930 bidirectional genes (some genes belonged to multiple pairs). We counted and performed Fisher's exact test between bidirectional genes with or without a TSS in ubi-PLSs (Figure 1D).

There were 1874 genes with TSSs overlapping both ubi-PLSs and non-ubi-PLSs (i.e. they have at least one TSS in ubi-PLSs and at least one TSS in non-ubi-PLSs). To investigate whether this number is significantly different from expected, we randomly assigned TSSs to be in ubi-PLSs while maintaining the numbers of TSSs in each gene and then counted the number of genes with TSSs overlapping both ubi-PLSs and non-ubi-PLSs.

The cell-essential gene data were obtained from previous publications (15–17). We counted the number of cell-essential genes (using the same cut-off as in each of the previous studies) that had ubi-PLSs or non-ubi-PLSs (Figure 1G). We also ranked all genes by their essentiality scores (15) and counted the percentage of genes with their TSSs overlapping ubi-PLSs by going down the ranks and examining each nonoverlapping 500-gene window. The percentage of all tested genes with TSSs overlapping ubi-PLSs is plotted as a control horizontal line (Figure 1H, Supplementary Figure S1C–F).

### Gene Ontology enrichment analysis (Figure 1E–F, and Supplementary Table S3)

We performed Gene Ontology enrichment analysis using the Panther tool (18) on the 9204 genes whose TSSs were located in ubi-PLSs (listed in Supplementary Table S2B). We used Fisher's exact test with a false discovery rate (FDR) correction and all *Homo sapien* genes as the background (Supplementary Table S3). We used the most enriched and depleted (FDR < 1.0 × 10^−10^) GO Biological Process terms to generate two word clouds with the R package *wordcloud* (Figure 1E, F).

### Normalized CG content (Figure 2A)

To evaluate whether cCRE-PLSs are enriched in CG dinucleotides, we used normalized CG content as previously described (19), defined as the ratio of the observed number over the expected number of CG dinucleotides, where the expected number is calculated as [(Fraction of C+Fraction of G)/2]^2^, i.e.

(1) $$\begin{eqnarray*}&&Normalized\ CG\ content\ \nonumber\\ &&\quad= \frac{{Fraction\ of\ CpG}}{{{{\left[ {\left( {Fraction\ of\ C + Fraction\ of\ G} \right)/2} \right]}^2}}}\ \end{eqnarray*}$$

**Figure 2.**
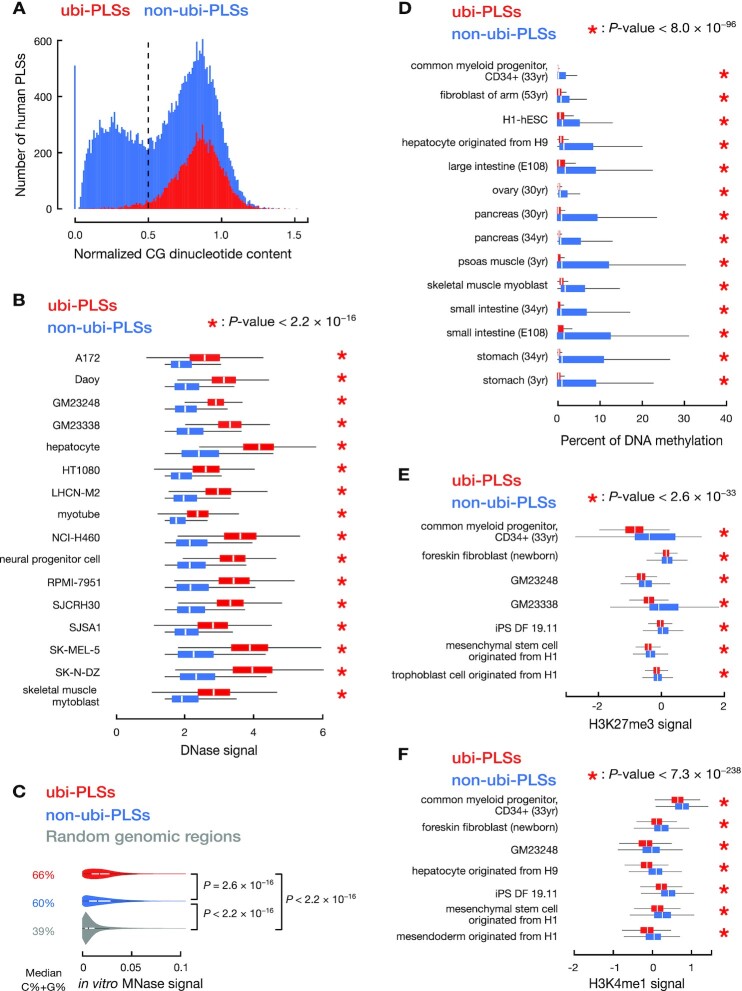
ubi-PLSs are enriched in CG dinucleotides and have open chromatin in cells but closed chromatin *in vitro*. (**A**) ubi-PLSs have higher CG dinucleotide content than non-ubi-PLSs. Histograms show that a vast majority of ubi-PLSs (red) have high CG dinucleotide content (> 0.5), while non-ubi-PLSs (blue) show a bimodal distribution of CG content. (**B**) ubi-PLSs (red) have significantly higher DNase signals than non-ubi-PLSs (blue) defined in the same biosample. Data for 16 biosamples are shown, with each biosample represented by a pair of boxplots. All *P*-values were computed with Wilcoxon rank-sum tests. (**C**) ubi-PLSs (red) have higher *in vitro* nucleosome occupancy (*in vitro* MNase-seq signals) than non-ubi-PLSs (blue). The *in vitro* MNase-seq experiment was performed on *in vitro* reconstructed nucleosomes using purified genomic DNA and recombinant histone proteins. We randomly selected 10 000 genome regions as control (gray). All *P*-values were computed using Wilcoxon rank-sum tests; *P*-values ≤ 2.2 × 10^−16^ for all comparisons. (**D**) Similar to Figure 2B, but for DNAme levels in 14 biosamples. (**E**) Similar to Figure 2B, but for H3K27me3 levels in seven biosamples. (**F**) Similar to Figure 2B, but for H3K4me1 levels in seven biosamples.

As reported previously (19), promoters show a bimodal distribution for their normalized CG content, with a valley at 0.5 (Figure 2A).

### Comparison of DNase, MNase, DNA methylation, RNA, RAMPAGE and histone mark levels between ubi-PLSs and non-ubi-PLSs (Figures 2B–F, 3A–B and Supplementary Figures S2, S4, S8)

By definition, ubi-PLSs have accessible chromatin in most biosamples while non-ubi-PLSs have accessible chromatin in only a few samples. To compare ubi-PLSs with non-ubi-PLSs at their chromatin and transcriptional levels, we defined non-ubi-PLSs for each biosample as those cCRE-PLSs that were not ubi-PLSs and yet had a high DNase signal in that sample.

To assess the chromatin accessibility of cCRE-PLSs, we compared the DNase signals between ubi-PLSs and biosample-specific non-ubi-PLSs. DNase-seq data on 16 biosamples were downloaded from the ENCODE portal (ENCODE accessions in Supplementary Table S1C) we selected these 16 biosamples because they also had RNA-seq and RAMPAGE data (see below). We performed Wilcoxon rank-sum tests between ubi-PLSs and non-ubi-PLSs (Figure 2B).

To assess the inherent nucleosome-forming tendencies of ubi-PLSs and non-ubi-PLSs, we reanalyzed the previously published *in vitro* MNase-seq data (GEO accession GSE25133) (20). The *in vitro* MNase-seq data was generated by performing MNase-seq experiments on *in vitro* reconstructed nucleosomes using purified genomic DNA and recombinant histone proteins H2A, H2B, H3, and H4 (20). We first mapped *in vitro* MNase-seq reads to GRCh38 using Bowtie v1.2.3 with parameters ‘-C -l 25 -n 2’ (21). We then shifted all mappable reads in the 3′ direction by 55 nucleotides, half of the average core nucleosome size, to center MNase signals on the nucleosome dyad. Finally, we compared *in vitro* MNase signals on ubi-PLSs versus non-ubi-PLSs and performed Wilcoxon rank-sum tests between the signal levels (Figure 2C). As control, we also included in Figure 2C 10 000 randomly selected genomic regions with the length 327 bp (the median length of cCRE-PLSs).

We compared the DNA methylation levels between ubi-PLSs and biosample-specific non-ubi-PLSs. Whole genome bisulfite sequencing data on 14 biosamples were downloaded from the ENCODE portal (ENCODE accessions in Supplementary Table S1I). We selected these 14 biosamples because they also had matched DNase-seq data (assayed on biosamples with the same donor ID). We performed Wilcoxon rank-sum tests between ubi-PLSs and non-ubi-PLSs (Figure 2D). Similarly, we compared the H3K27me3 (Figure 2E) and H3K4me1 (Figure 2F) signals between ubi-PLSs and biosample-specific non-ubi-PLSs in biosamples with data.

We made meta plots to compare the histone mark ChIP-seq signals between ubi-PLSs and non-ubi-PLSs in K562 cells. For all ubi-PLSs and K562 non-ubi-PLSs, we calculated the average ChIP-seq signals of eight histone marks and the average MNase-seq signal as a measure of nucleosome occupancy. We downloaded ChIP-seq and MNase-seq data from the ENCODE portal (ENCODE accessions in Supplementary Table S1F and S1G) and used the UCSC’s bigWigAverageOverBed tool to calculate the average signal in the ±2kb window centered on each group of cCRE-PLSs (Supplementary Figure S2).

**Figure 3.**
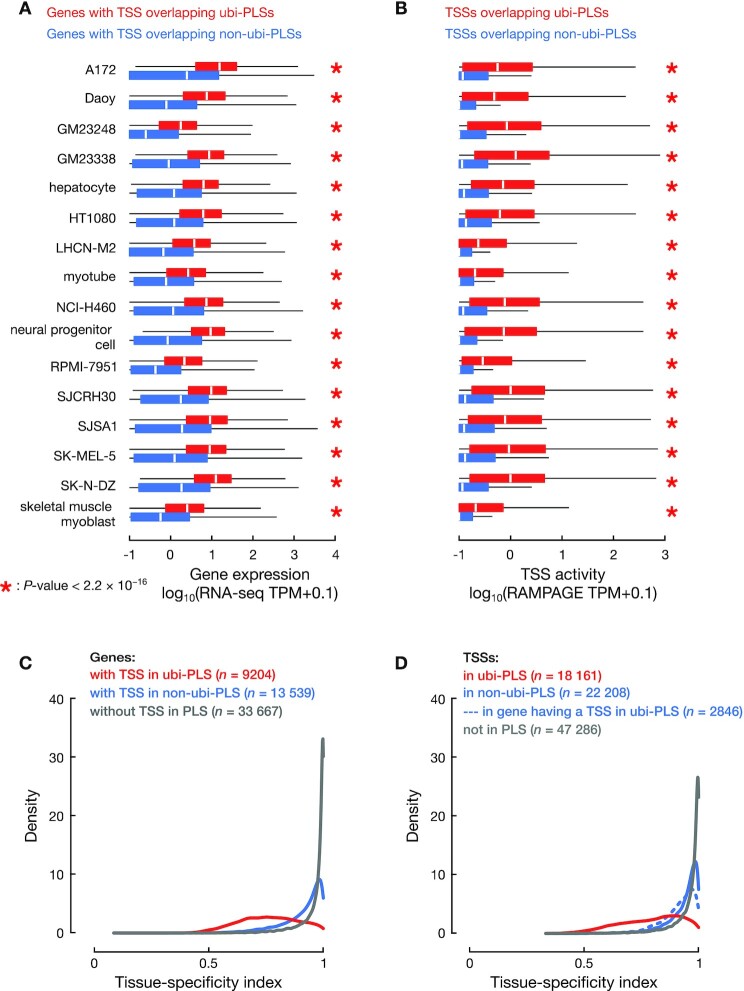
ubi-PLSs are promoters of ubiquitously expressed genes. (**A**) Genes whose TSSs overlapped ubi-PLSs (red) are significantly more highly expressed than genes whose TSSs overlapped non-ubi-PLSs (blue) in the same biosample. Expression levels were obtained from RNA-seq data, quantified by TPM (with a pseudocount of 0.1 added to each gene), and plotted in log scale. All *P*-values were computed with Wilcoxon rank-sum tests. (**B**) TSSs overlapping ubi-PLSs (red) are significantly more active than TSSs overlapping non-ubi-PLSs (blue) in the same biosample. TSS activities were computed using RAMPAGE data, quantified by TPM (with a pseudocount of 0.1 added to each TSS), and plotted in log scale. All *P*-values were computed with Wilcoxon rank-sum tests. (**C**) Distributions of the tissue-specificity index for genes whose TSSs overlap ubi-PLSs (red), overlap non-ubi-PLS (blue), and do not overlap a cCRE-PLS (gray). The tissue-specificity index was computed using RNA-seq data across 103 human biosamples (see Materials and Methods). The three distributions are significantly different (Wilcoxon rank-sum test *P*-values < 2.2 × 10^−16^ for ubi-PLS versus non-ubi-PLS or versus non-PLS). (**D**) Distributions of the tissue-specificity index for TSSs overlapping ubi-PLSs (red), TSSs of the genes without a ubi-PLS overlapping TSS but with at least one TSS overlapping a non-ubi-PLS (solid blue), other TSSs of the genes with at least one TSS overlapping a ubi-PLS (dashed blue), and TSSs not overlapping a cCRE-PLS (gray). All the distributions are significantly different (Wilcoxon rank-sum test *P*-values < 2.2 × 10^−16^ for TSSs overlapping ubi-PLS versus each of the other three groups of TSSs).

To compare the gene expression level and TSS activity between ubi-PLSs and biosample-specific non-ubi-PLSs, we downloaded RNA-seq and RAMPAGE data (ENCODE accessions in Supplementary Table S1D and S1E) in the aforementioned 16 biosamples with DNase-seq data from the ENCODE portal. We then compared the expression levels of genes (using RNA-seq data) and individual TSSs (using RAMPAGE data) associated with ubi-PLSs and non-ubi-PLSs in each of the 16 biosamples. We performed Wilcoxon rank-sum tests between ubi-PLSs and non-ubi-PLSs (Figure 3A, B). We also used the RNA-seq signal to compare the expression level of transcription factors with motif enrichment in ubi-PLSs versus non-ubi-PLSs (Supplementary Figure S8).

Using RNA-seq data in the aforementioned 16 biosamples, we specifically compared the expression levels of the transcripts whose TSSs overlapped ubi-PLSs and the expression levels of the transcripts whose TSSs overlapped non-ubi-PLSs in each biosample. For this analysis, we only used the 1874 genes with some TSSs overlapping ubi-PLSs and other TSSs overlapping non-ubi-PLSs. We performed Wilcoxon rank-sum tests between the two sets of transcripts in each biosample (Supplementary Figure S4A).

Using RNA-seq data in the A172 cell line, we compared the standard deviation and the mean of expression levels of the transcripts whose TSSs overlapped the same ubi-PLS (Supplementary Figure S4B, top). Likewise, we performed the analysis for the transcripts whose TSSs overlapped the same non-ubi-PLS defined in A172 (Supplementary Figure S4B, bottom).

### Chromatin interaction analysis (Supplementary Figure S3)

We downloaded ChIA-PET data (22) from the Gene Expression Omnibus (GEO) with the accession GSE72816. This dataset included RNA Pol II (RNAPII) and CTCF ChIA-PET clusters in HeLa and GM12878 cell lines provided in the human genome version hg19. We filtered each set of clusters by retaining the ChIA-PET loops that were supported by at least four reads. To compare with ChIA-PET clusters, we used the liftOver tool to map ubi-PLSs and non-ubi-PLSs from GRCh38 to hg19. We further resized all cCRE-PLSs to 345 bp (the median length of cCRE-PLSs in hg19) to eliminate the difference in the length distributions of the two sets of cCRE-PLSs (Supplementary Figure S1A). We then intersected the ChIA-PET loop anchors with ubi-PLSs and biosample-specific non-ubi-PLSs, requiring at least 1 bp overlap. We calculated the percentage of cCRE-PLSs that overlapped ChIA-PET loop anchors as well as the percentage of ChIP-PET loop anchors that overlapped cCRE-PLSs. Fisher's exact tests were performed between ubi-PLSs and non-ubi-PLSs (Supplementary Figure S3).

### Tissue-specificity index (Figures 3C, D and 5C)

We used a previously defined tissue-specificity index (23) to evaluate the tissue specificity of gene expression and TSS activities, defined as:

(2) $$\begin{equation*}Tissue - specificity\ index\ = \frac{1}{{N - 1}}\ \mathop \sum \limits_{i\ = \ 1}^N \left( {1 - {x_i}} \right)\end{equation*}$$

where $N$ is the number of biosamples and ${x_i}$ is the expression profile component normalized by the maximal component value across all biosamples. This tissue-specificity index ranges from 0 to 1, with a higher value indicating a higher degree of tissue specificity.

To calculate the tissue-specificity index, we used RNA-seq and RAMPAGE data from the ENCODE portal (ENCODE accessions in Supplementary Table S1D and E), encompassing 103 biosamples for which both types of data were available. Prior to computing the tissue specificity of genes or TSSs, we performed quantile normalization across genes or TSSs in each sample and then applied the above formula on the normalized RNA-seq and RAMPAGE values (Figure 3C, D). We also used the RNA-seq data to compute the tissue-specificity index for transcription factors (Figure 5C).

### The number of TSSs in cCRE-PLSs (Figure 4A)

**Figure 4.**
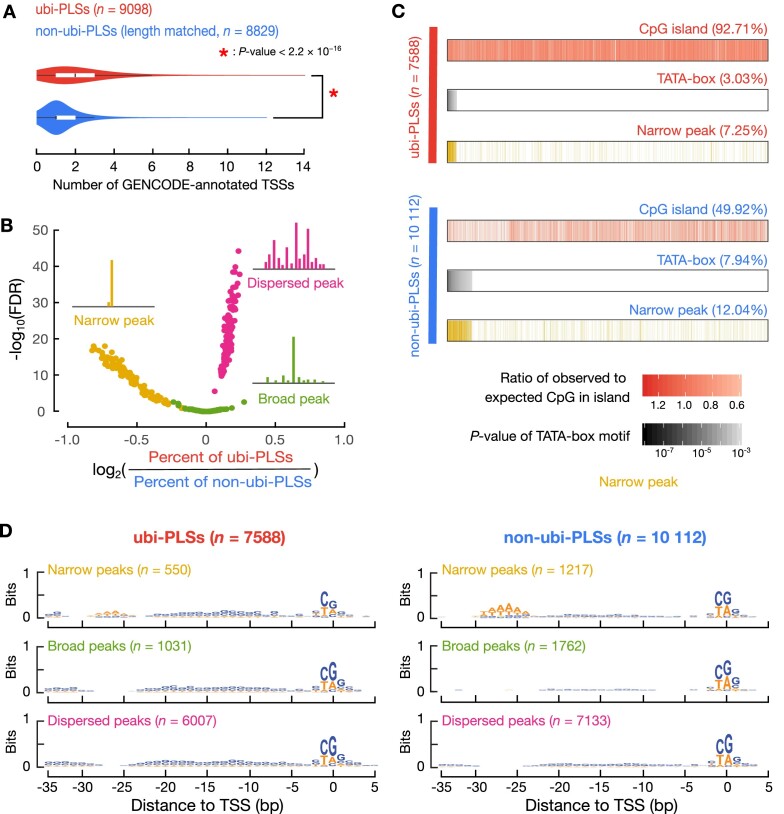
The promoter features of ubi-PLSs. (**A**) ubi-PLSs (red) tend to have multiple GENCODE-annotated TSSs while non-ubi-PLSs (blue) normally contain one TSS. A subset of non-ubi-PLSs were used to match the length distribution of ubi-PLSs. The *P*-value was computed with a Wilcoxon rank-sum test. (**B**) ubi-PLSs are enriched in dispersed peaks, while non-ubi-PLSs are enriched in narrow peaks. The three types of promoter peak shape (narrow, broad, dispersed) were defined using RAMPAGE data in 115 biosamples (see Materials and Methods). For each type of promoter peak shape, we generated a volcano plot with the x-axis showing the log_2_(fold change) of the percentage of ubi-PLSs over the percentage of non-ubi-PLSs assigned to a peak shape in a RAMPAGE dataset, and the y-axis showing the significance of the enrichment in that peak shape (-log_10_ of the Fisher's exact test *P*-values after FDR correction). We overlaid all three volcano plots for comparison (one yellow, one red, and one green, plotting the enrichment for narrow, dispersed, and broad peaks, respectively). (**C**) ubi-PLSs are more likely to overlap CpG islands and less likely to have TATA-boxes than non-ubi-PLSs. Two groups of bars with columns depict ubi-PLSs and non-ubi-PLSs, respectively. The top bar in each group shows the enrichment of CpG islands, the middle bar shows the enrichment of TATA-boxes, and the bottom bar shows whether a cCRE-PLS is a narrow peak. The ubi-PLSs and non-ubi-PLSs (individual columns in each bar) are sorted from left to right by the –log_10_(*P*-value of the TATA-box motif). (**D**) Sequence logos of ubi-PLSs and non-ubi-PLSs with narrow, broad, and dispersed peaks, respectively.

We used GENCODE-annotated TSSs (v24 basic, the same version used in ENCODE cCRE definition) (24). GENCODE-annotated TSSs of transcripts with inactive or uncertain biotypes such as pseudogene and TEC (to be experimentally confirmed) were removed. To remove the impact of the length difference between ubi-PLSs and non-ubi-PLSs, we randomly selected a subset (*n* = 8829) of non-ubi-PLSs to match the length distribution of ubi-PLSs (Supplementary Figure S1B). We intersected the genomic coordinates of TSSs with those of cCRE-PLSs and counted the number of TSSs overlapping each ubi-PLS and non-ubi-PLS in order to draw the violin-box plot (Figure 4A). A Wilcoxon rank-sum test was performed between ubi-PLSs and non-ubi-PLSs.

### Definition of RAMPAGE peak shape and calculation of the enrichment of cCRE-PLSs in rPeaks with each shape (Figure 4B–D, Supplementary Figure S5A)

We defined the shape of each of the 52 546 representative RAMPAGE peaks (rPeaks) (25) using RAMPAGE data in 115 biosamples downloaded from the ENCODE portal (ENCODE accessions in Supplementary Table S1E). Each rPeak was classified as one of three peak shapes according to the flowchart in Supplementary Figure S5A. Dispersed peaks were defined as rPeaks in which fewer than 50% of the RAMPAGE reads within that peak had their 5′-ends overlapping the region ±2 nt around the peak summit. The remaining rPeaks were further divided into narrow peaks or broad peaks according to peak length (broad peak, >9 nts; narrow peak, ≤9 nts). For each rPeak, we used the same boundary in all biosamples, but the peak summit position and peak shape were determined in each biosample (only for the rPeaks with at least 10 RAMPAGE reads in a sample), which showed small variations across the biosamples.

Using the rPeak shapes defined in 115 biosamples, we computed the enrichment of cCRE-PLSs in the rPeaks with each shape type as follows. We first calculated the fold change between the number of ubi-PLSs and the number of biosample-specific non-ubi-PLSs that overlapped the rPeaks with each shape in each biosample. We then performed a Fisher's exact test for each biosample and each peak shape, followed by FDR correction of the resulting *P*-value. Finally, we make a volcano plot using the fold change and *P*-value for each rPeak shape, and overlay the three volcano plots together for comparison (Figure 4B).

For cCRE-PLSs with RAMPAGE peaks in at least one of the 115 biosamples, we tested whether a cCRE-PLS overlapped a CpG island (26), with CpG island annotations downloaded from the UCSC Genome Browser (hg38, cpgIslandExtUnmasked.txt). We then took the ratio of the observed over the expected numbers of CpG dinucleotides in each cCRE-PLS from this annotation file (Figure 4C). We also tested whether a cCRE-PLS contained a TATA-box by looking for a site for the TATA-box motif within 25–35 bp upstream of the TSS, with the TSS defined by the summit of RAMPAGE peaks. TATA-box sites were predicted using the FIMO algorithm (27) with the parameters --norc --thresh 1e-3 and the TBP position-weight matrix (MA0108.2) from the JASPAR database (28) (Figure 4C).

We grouped ubi-PLSs and non-ubi-PLSs according to the shape of rPeaks that they overlapped in the most biosamples, and created sequence logos for each cCRE-PLS sub-group using WebLogo 3 (29) (Figure 4D).

### Transcription factor motif and ChIP-seq peak analysis (Figures 5A–B and 6D, Supplementary Table S4 and Supplementary Figures S6 and S7)

**Figure 5.**
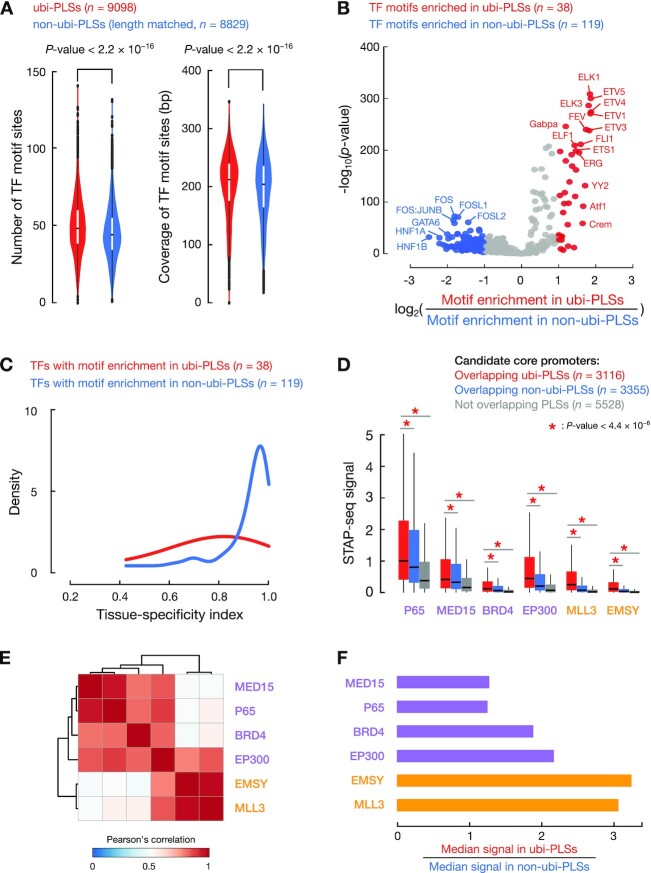
ubi-PLSs have more transcription factor binding sites than non-ubi-PLSs, and the two sets of cCRE-PLSs are enriched in different transcription factor motifs. (**A**) Violin plots compared the number of transcription factor binding sites (left) and the total number of genomic positions covered by transcription factor binding sites (coverage, right) between ubi-PLSs (red) and non-ubi-PLSs (blue). Non-ubi-PLSs were downsampled to match the length distribution of ubi-PLSs for a fair comparison. All *P*-values were computed with Wilcoxon rank-sum tests. (**B**) The volcano plot shows the enriched transcription factor motifs for ubi-PLSs versus non-ubi-PLSs. Each dot represents a transcription factor motif. The x-axis shows the log_2_(fold change) of the transcription factor motif enrichment in ubi-PLSs over non-ubi-PLSs, and the y-axis depicts Fisher's exact test *P*-value of the enrichment. Transcription factors whose motifs are enriched in ubi-PLSs are in red, while transcription factors whose motifs are enriched in non-ubi-PLSs are in blue. (**C**) Transcription factors that prefer to bind ubi-PLSs are less tissue-specific than the transcription factors that prefer to bind non-ubi-PLSs. The two groups of transcription factors are defined by their enriched motifs in panel B, and the tissue-specificity index was calculated using RNA-seq data across 103 samples as in Figure 3C. TF: transcription factor. (**D**) Boxplots show the activities (STAP-seq signals) of the candidate core promoters that overlapped ubi-PLSs (red), overlapped non-ubi-PLSs (blue), or did not overlap cCRE-PLSs (gray). STAP-seq data on six transcriptional cofactors were available and the colors of the cofactors represent their preference for regulating TATA-box (purple) or CG-rich (orange) promoters. All *P*-values were computed with Wilcoxon rank-sum tests. (**E**) Hierarchical clustering of the six cofactors based on Pearson's correlation of STAP-seq signals across the 6471 promoter candidates overlapping cCRE-PLSs. (**F**) Ratios of the median STAP-seq signals for promoter candidates overlapping ubi-PLSs versus those overlapping non-ubi-PLSs.

**Figure 6.**
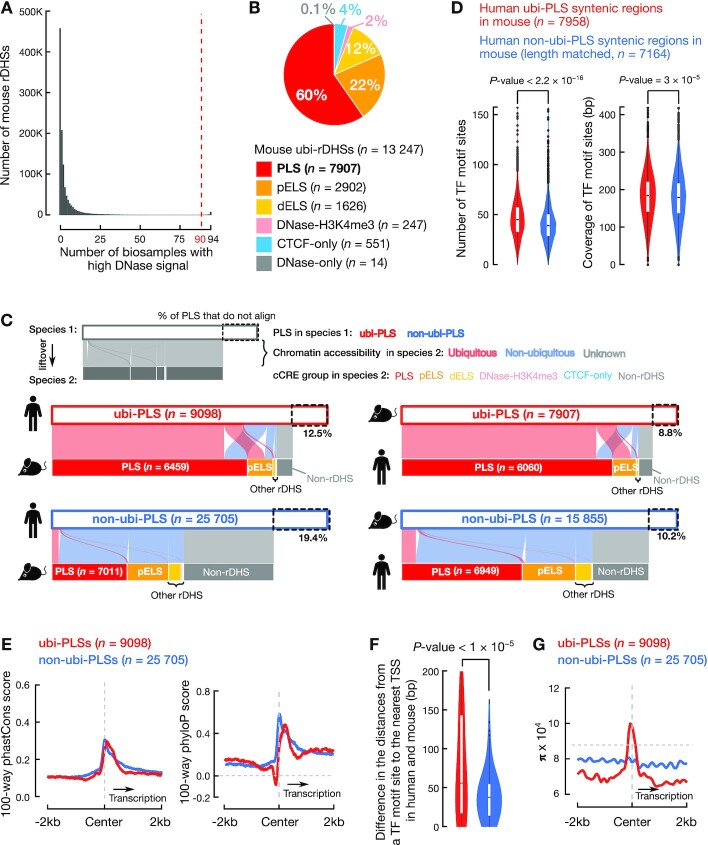
Human ubi-PLSs are conserved in mouse. (**A**) Definition of mouse ubi-rDHSs. A histogram shows the number of mouse rDHSs that have high DNase signals in a certain number of biosamples. rDHSs that have high DNase signals in 90 or more biosamples (out of a total of 94 biosamples) are defined as ubi-rDHSs in mouse. (**B**) The pie chart shows that 82% of mouse ubi-rDHSs are cCRE-PLSs or cCRE-pELSs. The category of ubi-rDHSs are as in Figure 1B. (**C**) Most human ubi-PLSs are also mouse ubi-PLSs and vice versa. The two alluvial plots on the left show the syntenic regions of human cCRE-PLSs in the mouse genome, and the two alluvial plots on the right show the syntenic regions of mouse cCRE-PLSs in the human genome. The color of a ribbon indicates whether a cCRE-PLS is ubiquitous (pink) or not (light blue) in the other genome, while a gray ribbon indicates that although some cCRE-PLSs can be lifted over to the other genome, they are no longer rDHSs in that other genome. (**D**) Human ubi-PLSs maintain their higher density and diversity of transcription factor binding sites in the mouse genome. As in Figure 4A, mouse regions that are lifted over from human ubi-PLSs (red) have more transcription factor binding sites and higher transcription factor binding site coverage than the mouse regions lifted over from human non-ubi-PLSs (blue). All *P*-values were computed with Wilcoxon rank-sum tests. TF: transcription factor. (**E**) Average 100-way phastCons scores (left) and phyloP scores (right) of the ±2 kb genomic regions centered on human ubi-PLSs (red) and non-ubi-PLSs (blue). The horizontal dashed gray line denotes the background phyloP score of zero. The vertical dashed gray line indicates the center of cCRE-PLSs. (**F**) Violin-box plots show the distributions of the difference between human and mouse in the distance from transcription factor motif sites in ubi-PLSs (red) and non-ubi-PLSs (blue) to the nearest TSS. The Wilcoxon rank-sum test *P*-value is shown. TF: transcription factor. (**G**) Average nucleotide diversity (π) in the ±2 kb genomic regions centered on human ubi-PLSs (red) and non-ubi-PLSs (blue). The horizontal dashed gray line represents the genome-wide background level of π. The vertical dashed gray line indicates the center of cCRE-PLSs.

We found that ubi-PLSs were slightly longer than non-ubi-PLSs (median lengths are 349 and 295 bp, respectively; Supplementary Figure S1A). To avoid the impact of the length difference on transcription factor motif analysis, we used the subset of non-ubi-PLSs (*n* = 8829) that matched the length distribution of ubi-PLSs as described above for Figure 5A (Supplementary Figure S1B). We scanned the two sets of cCRE-PLSs for motif matches using FIMO (27) with the parameters --thresh 1e-4 and the position-weight matrices downloaded from the JASPAR database (28). We counted the number of transcription factor motif sites as well as the total number of genomic positions covered by these motif sites in each cCRE-PLS. Wilcoxon rank-sum tests were performed to compare ubi-PLSs and non-ubi-PLSs (Figure 5A). We performed the same analysis on the syntenic regions of the human ubi-PLSs and non-ubi-PLSs in the mouse genome (Figure 6D). For each transcription factor, we compared the enrichment of its motif between ubi-PLSs and non-ubi-PLSs by both fold enrichment and Fisher's exact test in a volcano plot (Figure 5B). Transcription factors with at least two-fold enrichment for their motif sites in ubi-PLS vs. non-ubi-PLS or vice versa were identified (Supplementary Table S4).

Furthermore, we extended the transcription factor motif enrichment analysis in a specific biosample by including the transcription factor ChIP-seq data in the same biosample, namely, HepG2, H1, K562, or GM12878 cells. For transcription factors with matching position-weight matrices and ChIP-seq data in one of these four cell lines, we downloaded transcription factor ChIP-seq peaks from the ENCODE portal (ENCODE accessions in Supplementary Table S1H). A cCRE-PLS overlapping a transcription factor ChIP-seq peak (by at least half of the peak width) in a cell line and containing a site for the transcription factor motif was considered to be bound by the transcription factor in that cell line. We measured the enrichment by both fold enrichment and Fisher's exact test in each cell line (Supplementary Figure S6).

We made aggregation plots to evaluate the transcription factor ChIP-seq signal on cCRE-PLSs. We downloaded the ChIP-seq signals (bigWig files) of six transcription factors from the ENCODE portal (ENCODE accessions in Supplementary Table S1H), with three showing a preference for ubi-PLSs and the other three showing a preference for non-ubi-PLSs (Figure 5B). We used UCSC’s bigWigAverageOverBed tool to calculate the average signal across the ±2 kb window centered on cCRE-PLSs (Supplementary Figure S7).

### STAP-seq analysis (Figure 5D–F)

We analyzed the promoter activities of ubi-PLSs and non-ubi-PLSs using the STAP-seq data produced using a high-throughput promoter assay (GEO accession GSE126221) (30). Because the STAP-seq data were in human genome version hg19, we lifted the genomic coordinates of cCRE-PLSs in GRCh38 to hg19 using the chain files and the liftOver method from the UCSC Genome Browser. We overlapped the 11 979 candidate core promoters tested using the STAP-seq assay in the human HCT116 cell line with our two sets of cCRE-PLSs. We then compared the STAP-seq signals among three groups of candidate core promoters: those that overlapped ubi-PLSs (*n* = 3116), those that overlapped non-ubi-PLSs (*n* = 3335), and those that did not overlap any cCRE-PLSs (*n* = 5528; Figure 5D). We performed hierarchical clustering of the cofactors based on Pearson's correlation of STAP-seq signals across the 6451 cCRE-PLS-overlapping candidate core promoters (Figure 5E). We then calculated the ratio between the median STAP-seq signal of the candidate core promoters that overlapped ubi-PLSs versus non-ubi-PLSs for each cofactor (Figure 5F).

### Evolutionary conservation and enrichment in Mendelian-disease genes (Figures 6C, E–F and 7A–B and Supplementary Figure S9F, G)

We mapped human cCRE-PLSs to the mouse genome (mm10) using the chain files and the liftOver method from the UCSC Genome Browser, with the parameter -minMatch = 0.5. We then asked whether these syntenic regions were also cCREs in the mouse genome. A syntenic region in the mouse was considered a cCRE when at least half of the region overlapped a mouse cCRE. These syntenic regions overlapping mouse cCREs were regarded as ubiquitous or not according to the mouse cCREs (Figure 6C). We performed a similar analysis to map the mouse cCRE-PLSs to the human genome.

We downloaded the human 100-way phastCons and phyloP signals (bigWig files) from the UCSC Genome Browser. We used UCSC’s bigWigAverageOverBed tool to calculate the average signal across the ±2 kb windows centered on cCRE-PLSs (Figure 6E).

To test whether there is a shift in transcription factor motif sites between human and mouse, we measured the distance from each motif site to the nearest GENCODE-annotated TSS (v24 basic for human and vM18 basic for mouse). We included in this analysis only those transcription factor motif sites with significant FIMO *P*-values (<1 × 10^−5^) in both human and mouse genomes. We only included in this analysis the human cCRE-PLSs that could be lifted over to the mouse genome and their syntenic regions. Wilcoxon rank-sum tests were used to compare the distances between human and mouse (Figure 6F).

**Figure 7.**
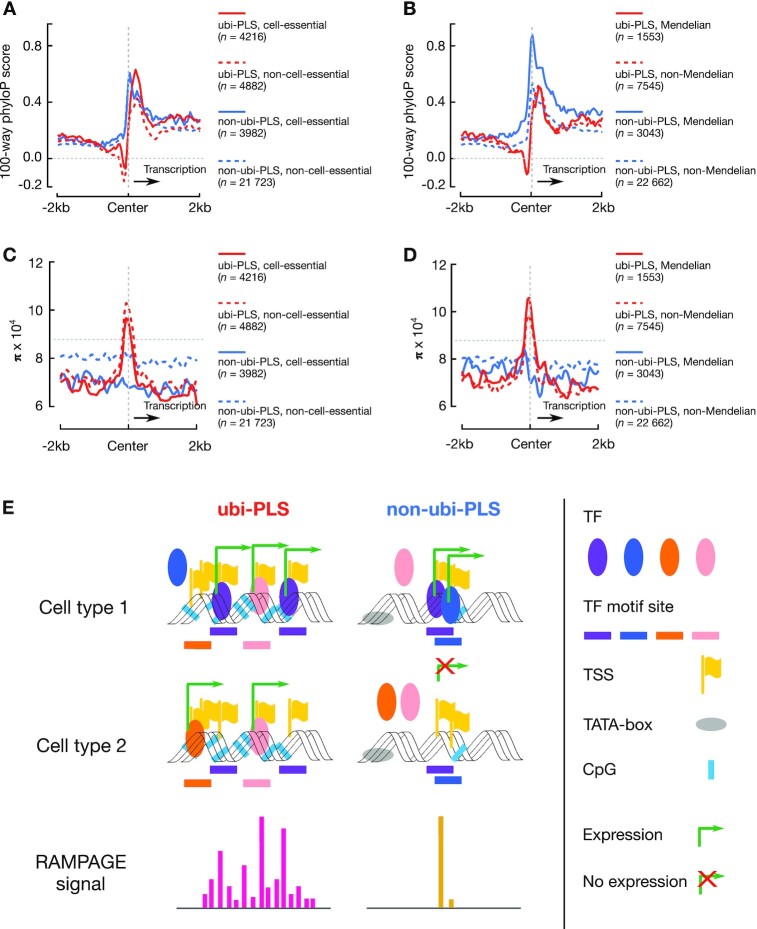
Evolution conservation and human variation around ubi-PLSs and non-ubi-PLSs and a schematic model of transcriptional activation for the two groups of cCRE-PLSs. (**A**) Average 100-way phyloP scores in the ±2 kb genomic regions centered on human ubi-PLSs (red) and non-ubi-PLSs (blue), each subdivided into cell-essential (solid line) and non-cell-essential (dashed line) subsets. The horizontal dashed gray line represents the genome-wide background and the vertical dashed gray line indicates the center of the cCRE-PLSs in all panels A–D. (**B**) Average 100-way phyloP scores in the ±2 kb genomic regions centered on human ubi-PLSs (red) and non-ubi-PLSs (blue), each subdivided into Mendelian-disease (solid line) and non-Mendelian-disease (dashed line) subsets. (**C**) Average nucleotide diversity (π) in the ±2 kb genomic regions centered on human ubi-PLSs (red) and non-ubi-PLSs (blue), each subdivided into cell-essential (solid line) and non-cell-essential (dashed line) subsets. (**D**) Average nucleotide diversity (π) in the ±2 kb genomic regions centered on human ubi-PLSs (red) and non-ubi-PLSs (blue), each subdivided into Mendelian-disease (solid line) and non-Mendelian-disease (dashed line) subsets. (**E**) ubi-PLSs tend to have high CG content, lack a TATA-box, be bound by multiple transcription factors, and initiate transcription from dispersed genomic positions. They recruit different transcription factors to initiate transcription in different cell types, maintaining high and ubiquitous expression. In contrast, non-ubi-PLSs are more likely to have lower CG content, contain a TATA-box, be bound by fewer transcription factors, and initiate transcription from a specific genomic position. They are expressed in a few cell types and controlled by a select group of transcription factors. TF: transcription factor; TSS: transcription start site.

We merged all cell-essential genes from three CRISPR screen studies (15–17) and arrived at a list of 5312 cell-essential genes. In total, these three studies investigated 18 633 genes. We defined a cCRE-PLS as cell-essential if its center is within 200 bp of the TSS of a cell-essential gene. We used UCSC’s bigWigAverageOverBed tool to calculate the average signal across the ±2 kb windows centered on the cell-essential and non-cell-essential subsets of ubi-PLSs and non-ubi-PLSs (Figure 7A).

We downloaded a list of Mendelian disease genes from the Online Mendelian Inheritance in Man (OMIM) resource (31) (genemap2.txt, accessed on 9 December 2020), requiring each gene to be associated with at least one phenotype that was not in brackets (non-diseases) or braces (multifactorial disorders), arriving at 2759 Mendelian-disease genes. Most (*n* = 2718) of these 2759 Medelian-disease genes were among the 18 633 genes investigated by the CRISPR screen studies (15–17), and we performed a Fisher's exact test on the overlap between the 2718 Mendelian-disease genes and the 5312 cell-essential genes with the total number of genes being 18 633. We assigned a cCRE-PLS to be in the Mendelian-disease group if its center was within 200 bp of the TSS of a Mendelian-disease gene. We further analyze the conservation score by using UCSC’s bigWigAverageOverBed tool to calculate the average signal across the ±2 kb windows centered on cCRE-PLSs. (Figure 7B). We used the top 100 Mendelian diseases that are the most frequently associated with ubi-PLSs or non-ubi-PLSs to generate two word clouds with the R package *wordcloud* (Supplementary Figure S9F, G). We also compared the frequency of the Mendelian-disease genes whose TSSs overlap non-ubi-PLSs (*n* = 1780 genes) that are implicated in each Mendelian disease with the frequency of the Mendelian-disease genes whose TSSs overlap ubi-PLSs (*n* = 784 genes) that are implicated in the same Mendelian disease (Supplementary Figure S9H).

### Human variation analysis (Figures 6G and 7C–D, and Supplementary Figure S9A–E)

To test the genetic variation in human populations, we calculated nucleotide diversity (**π**) using the following equation:

(3) $$\begin{equation*}\pi \ = \ 1 - \mathop \sum \limits_{i\ = \ 1}^n p_i^2\end{equation*}$$

where ${p_i}$ is the allele frequency of the *i-*th allele at a genomic position and *n* is the total number of alleles at that position. We used the human variation data from the NHLBI TOPMed project (32) (freeze 8 VCF files were downloaded from the Bravo web server: https://bravo.sph.umich.edu/freeze8/hg38/). Only variants passing all filters (‘PASS’) were considered. Indels were removed from all analysis. We averaged π across the genomic positions in each cCRE-PLS (π = 0 for positions without variants) to arrive at one π value for the cCRE-PLS. Similarly, we also computed π for the 2 kb upstream and the 2 kb downstream regions flanking cCRE-PLSs, with the direction of transcription oriented downstream (Figures 6G, 7C–D). To quantify the statistical difference between two groups of regions, we performed 10 000 bootstraps on the π values of the two groups of regions and computed the t-statistic for each bootstrap. The empirical *P*-value is then the fraction of bootstraps whose t-statistic values exceed the actual t-statistic value of the two groups. Supplementary Figure S9A–E shows the mean, the 95th percentile confidence interval, and *P*-value calculated by bootstrapping.

## RESULTS

### Three-quarters of ubiquitously DNase-accessible sites are proximal to TSSs

The 2 157 387 human rDHSs previously defined by the ENCODE consortium (12) showed a bimodal distribution in terms of the number of biosamples in which they had high DNase-seq signals (Figure 1A, Supplementary Table S1A). Most of these rDHSs had high DNase signals in one or a few biosamples, while a small but distinct subset of rDHSs had high DNase-seq signals in almost all of the 517 human biosamples included in our study (Figure 1A). We defined the 15 989 rDHSs with high DNase signals in 500 or more biosamples as ubi-rDHSs (Supplementary Table S2A).

ENCODE deemed 926 535 rDHSs—those with high signals of two histone modifications (H3K4me3 and H3K27ac, characteristic of promoters and enhancers, respectively) (33,34) or the chromatin-structure protein CTCF—as cCREs, and further classified these cCREs into five groups (12). Nearly all ubi-rDHSs were cCREs (Figure 1B), and 57% of ubi-rDHSs (*n* = 9098) were cCREs with a promoter-like signature (PLS; high H3K4me3 and having a TSS within 200 bp of the rDHS center) (12). Additionally, 24% of ubi-rDHSs (*n* = 3885) were TSS-proximal cCREs with an enhancer-like signature (pELS; high H3K27ac and having a TSS within 2000 bp of the rDHS center) (12). In aggregate, most ubi-rDHSs were within 500 bp of a TSS (Figure 1C). We focused our subsequent analyses on the 9098 ubi-rDHSs that were also cCRE-PLSs, which we denote ubi-PLSs. For comparison, we refer to the remaining 25 705 cCRE-PLSs that were not ubi-rDHSs as non-ubi-PLSs. While all cCREs are 150–350 bp long, ubi-PLSs are slightly longer than non-ubi-PLSs (Supplementary Figure S1A). To account for the length difference in some of our analyses (e.g. motif enrichment), we randomly selected a subset of non-ubi-PLSs with a matched length distribution (Supplementary Figure S1B).

### ubi-PLSs define essential genes

The 9098 ubi-PLSs overlapped 18 161 GENCODE V24 annotated TSSs, which belonged to 9204 genes (Supplementary Table S2B and C). Among these, 7330 genes have TSSs exclusively overlapping ubi-PLSs, and the other 1874 genes additionally have one or more TSSs overlapping non-ubi-PLSs. Meanwhile, 13 543 genes have TSSs only overlapping non-ubi-PLSs. The number of genes with both TSSs overlapping ubi-PLSs and other TSSs overlapping non-ubi-PLSs (1874) is substantially lower than expected (6727 ± 44, *P*-value < 1 × 10^−4^) if the TSSs were to be distributed randomly while maintaining the number of TSSs in each gene (see Materials and Methods). Thus, a distinct set of genes use ubi-PLSs to drive their transcription.

Furthermore, ubi-PLSs are enriched for the TSSs of protein-coding genes and bidirectional genes. The vast majority of ubi-PLSs contain the TSSs of protein-coding genes—7868 (85%) of the 9204 genes with TSSs overlapping ubi-PLSs are protein-coding genes (defined by GENCODE v24 basic, see Materials and Methods). Reciprocally, 40% of protein-coding genes have TSSs overlapping ubi-PLSs, while only 4% of non-protein-coding genes have TSSs overlapping ubi-PLSs (Fisher's exact test *P*-value < 2.2 × 10^−16^; Figure 1D). Among the 8930 bidirectional genes (defined in Materials and Methods), 4265 (48%) have TSSs overlapping ubi-PLSs (Figure 1D). Reciprocally, 46% of the 9204 genes with TSSs overlapping ubi-PLSs are bidirectional (Fisher's exact test *P*-value < 2.2 × 10^−16^).

Gene ontology analysis revealed that the 9204 genes with TSSs overlapping ubi-PLSs were enriched in universal biological processes such as various metabolic, biosynthetic, biogenesis and translational processes, but were depleted in specialized biological processes such as signaling, communication, sensory perception, response to stimuli and adaptive immune response (Figure 1E and F, Supplementary Table S3). Thus, ubi-PLSs correspond to the promoters of housekeeping genes that perform day-to-day cellular functions.

Cell-essential genes in the human genome were recently defined in several CRISPR screens (15–17). One study tested 18 166 genes in four cell lines, ranked these genes by their CRISPR scores in each cell line, and deemed the top 10% of the ranked genes essential (15). We found that 80–86% of these essential genes have TSSs overlapping ubi-PLSs (Figure 1G). Furthermore, when we ranked genes by essentiality (CRISPR scores in the range of –5.8 to 2.1 across the four cell lines; the more negative a score, the more essential the gene), we found that the percentages of genes overlapping ubi-PLSs decreased with the rank (Figure 1H). Two genes with TSSs overlapping ubi-PLSs, *RPL23A* (Ribosomal Protein L23a) and *CDC16* (cell division cycle 16), were deemed essential in all four tested cell lines (CRISPR scores –3.4 to –5.1 for *RPL23A* and –2.6 to –4.9 for *CDC16*). RPL23A is a component of the ribosome, responsible for protein synthesis. CDC16 is a protein ubiquitin ligase in the APC complex, which governs exit from mitosis via targeting cycle proteins for degradation by the 26S proteasome. Abnormal expression of *CDC16* can lead to diseases such as deafness and early infantile epileptic encephalopathy (35–37). We also tested the cell-essential genes defined in two other studies (16,17); 77–89% of essential genes defined in individual cell lines and 89–91% core essential genes (essential in multiple cell lines defined by each study) have TSSs overlapping ubi-PLSs (Figure 1G, Supplementary Figure S1C–F). Reciprocally, of the 9204 genes with a TSS overlapping a ubi-PLS, 3585 (40.0%) are cell essential (in total there are 5312 cell-essential genes in the three studies). Moreover, we found that the number of biosamples with high DNase signal is moderately correlated with the essentiality score (using CRISPR score in Wang *et al.*; Pearson correlation coefficient *r* = –0.24 for all cCRE-PLSs; *r* = –0.15 and –0.08 for ubi-PLSs and non-ubi-PLSs separately). In summary, ubi-PLSs regulate the transcription of genes that maintain essential cellular functions.

### ubi-PLSs are enriched in CG dinucleotides and their epigenetic environments are highly conducive to active transcription

Many promoter sequences are enriched in CG dinucleotides, and high-CG promoters tend to be constitutively expressed across cell types (19,38). The vast majority of ubi-PLSs have high CG dinucleotide content (normalized CG dinucleotide content ≥ 0.5) (19), while the non-ubi-PLSs show a bimodal distribution (Figure 2A). Accordingly, 89% of ubi-PLSs (8131 of 9098) overlap CpG islands, which is significantly higher than for non-ubi-PLSs (45%, 11 603 of 25 705, Fisher's exact test *P*-value < 2.2 × 10^−16^). Moreover, 510 out of 25 705 non-ubi-PLSs had zero CpG sites (hence CG dinucleotide content = 0 in Figure 2A), while none of ubi-PLSs had zero CpG sites. The high CG dinucleotide content of ubi-PLSs suggests that these promoters would support strong and ubiquitous transcription programs.

The DNase-seq signals at ubi-PLSs were substantially higher than those at non-ubi-PLSs (Figure 2B; 31–67% higher by median; Wilcoxon rank-sum test *P*-values < 2.2 × 10^−16^). We surveyed 16 human biosamples (see Materials and Methods; Supplementary Table S1C) and only used the subset of non-ubi-PLSs defined in each biosample for comparison with all ubi-PLSs. Accordingly, ubi-PLSs had lower nucleosome occupancy levels and more strongly positioned flanking nucleosomes than non-ubi-PLSs based on MNase-seq data in K562 cells (Supplementary Figure S2). We further examined the inherent nucleosome-forming tendencies for the two sets of cCRE-PLSs using *in vitro* MNase-seq data, that is, data from MNase-seq experiments performed on *in vitro* reconstituted nucleosomes using purified genomic DNA and recombinant core histone proteins (39). ubi-PLSs have a higher tendency to form nucleosomes *in vitro* than non-ubi-PLSs, and both sets of cCRE-PLSs have much higher tendencies than randomly selected genomic regions (Figure 2C). This finding is consistent with their relative G/C mononucleotide percentages (median 66%, 60% and 39% for ubi-PLSs, non-ubi-PLSs, and random genomic regions, respectively; Wilcoxon rank-sum test *P*-values < 2.2 × 10^−16^ for all three pairwise comparisons). A higher percentage of G/C mononucleotides facilitates nucleosome formation (20,39). Thus, ubi-PLSs take on the open chromatin status in cells despite having G/C rich sequences, which facilitate nucleosome formation (as shown by the *in vitro* MNase-seq experiment), and this open chromatin status in cells is due to trans-factors, which compete with histone proteins for access to the DNA in ubi-PLSs.

Next, we compared the level of DNA methylation and the signals of eight histone modifications around ubi-PLSs and non-ubi-PLSs. All cCRE-PLSs defined in a biosample have low DNA methylation levels, yet almost all ubi-PLSs have zero DNA methylation, showing significantly lower DNA methylation levels than non-ubi-PLSs defined in the corresponding biosample (Figure 2D, Wilcoxon rank-sum test *P*-values < 8.0 × 10^−96^ in all biosamples). Consistent with the DNase and MNase results described above, the levels of all histone marks examined were lower at the centers of ubi-PLSs than non-ubi-PLSs (Supplementary Figure S2 shows data in K562 cells), indicating that ubi-PLSs adopt more open chromatin structures than non-ubi-PLSs. For the flanking regions, especially the downstream regions with respect to the transcriptional direction (indicated by arrows pointing to the right in Supplementary Figure S2), the signals of promoter-enriched histone marks (H3K4me3, H3K4me2, H3K9ac, and H3K27ac) and transcription-induced histone mark H3K36me3 were substantially higher for ubi-PLSs than non-ubi-PLSs. However, this is the opposite for the repressive histone mark H3K27me3 and the enhancer-enriched mark H3K4me1 (Supplementary Figure S2). Comparisons of H3K27me3 and H3K4me1 signals in additional biosamples are provided in Figure 2E–F. Thus, ubi-PLSs have a more conducive epigenetic environment than non-ubi-PLSs for transcriptional output.

We further compared the activity of ubi-PLSs and non-ubi-PLSs using ChIA-PET data (22). Using four sets of ChIA-PET data (RNA Pol II and CTCF in HeLa and GM12878), we tested whether ubi-PLSs were more enriched in ChIA-PET loop anchors than non-ubi-PLSs. We observed a significantly higher percentage of ubi-PLSs located at loop anchors than non-ubi-PLSs (Fisher's exact test *P*-values < 2.2 × 10^−16^ for all four ChIA-PET datasets; Supplementary Figure S3A). Reciprocally, we also observed a higher percentage of loop anchors that overlapped ubi-PLSs than non-ubi-PLSs (Supplementary Figure S3B). These results suggest that ubi-PLSs are engaged in more three-dimensional chromatin interactions than non-ubi-PLSs.

### ubi-PLSs have higher transcription levels and display more ubiquitous expression profiles than non-ubi-PLSs

To directly quantify the transcriptional activity of ubi-PLSs, we analyzed RNA-seq and RAMPAGE data in the same 16 biosamples that we analyzed the DNase-seq data above (see Materials and Methods). RNA-seq data revealed that genes with at least one TSS overlapping ubi-PLSs (*n* = 9204) had significantly higher expression levels than genes with TSSs overlapping only non-ubi-PLSs in a specific biosample (Figure 3A; 3.8–12.4-fold higher by median; Wilcoxon rank-sum test *P*-values < 2.2 × 10^−16^). Among the 9204 genes, 5748–7817 (62–85%) were expressed with at least 1 transcript per million reads (TPM) in individual biosamples. When examined at the individual TSS level using RAMPAGE data, TSSs overlapping ubi-PLSs had substantially higher activity levels than TSSs overlapping non-ubi-PLSs in the same biosample (Figure 3B; 14.5-fold or higher by median; Wilcoxon rank-sum test *P*-values < 2.2 × 10^−16^). These results show that ubi-PLSs correspond to the promoters of highly expressed genes. We specifically examined the expression levels of the transcripts of 1874 genes with some TSSs overlapping ubi-PLSs and other TSSs overlapping non-ubi-PLSs. As expected, the transcripts with TSSs overlapping ubi-PLSs have higher expression levels than the transcripts with TSSs overlapping non-ubi-PLSs defined in the corresponding biosample (Supplementary Figure S4A; 2.4–15.5-fold higher by median, Wilcoxon rank-sum test *P*-values < 3.1 × 10^−11^). Overall, our results indicate that ubi-PLSs have higher transcription levels than non-ubi-PLSs.

We further investigated when a cCRE-PLS overlaps the TSSs of multiple transcripts whether these transcripts were expressed at similar levels. Using the RNA-seq data in neuronal progenitor cells, we plotted the standard deviation vs. the mean of expression levels of the transcripts whose TSSs overlap the same ubi-PLS (Supplementary Figure S4B). Although the TSSs that overlap the same ubi-PLS are often all active, there is a range of variations in their expression levels, suggesting that other factors besides open chromatin, such as transcription factor binding, also contribute to their activities. We performed the same analysis on transcripts whose TSSs overlap the same non-ubi-PLS, and they also show variation in expression levels, although as expected they are expressed at much lower levels than the transcripts whose TSSs overlap ubi-PLSs (Supplementary Figure S4B). We observed the same patterns for other biosamples (figures not shown).

Because promoters with high CG dinucleotide content tend to be ubiquitously expressed, and because ubi-PLSs are enriched in CG dinucleotides, we next evaluated the tissue specificity of ubi-PLSs by surveying 103 ENCODE biosamples with both RNA-seq and RAMPAGE data (for evaluation at the gene and TSS levels, respectively). Using a tissue-specificity index (23), genes with one or more TSSs overlapping ubi-PLSs were substantially less tissue-specific than genes with one or more TSSs overlapping non-ubi-PLSs but none overlapping ubi-PLSs (Figure 3C; Wilcoxon rank-sum test *P*-value < 2.2 × 10^−16^; median = 0.76 and 0.94, respectively). Both of these groups were less tissue-specific than the genes whose TSSs did not overlap any cCRE-PLSs (Figure 3C; Wilcoxon rank-sum test *P*-value < 2.2 × 10^−16^; median = 0.99 for genes with no TSSs overlapping cCRE-PLS), presumably because genes in the latter group were not expressed in the large number of biosamples assayed by ENCODE, consistent with their high tissue specificity. With the tissue-specificity index (23) computed using RAMPAGE data for individual TSSs, the TSSs overlapping ubi-PLSs showed much lower tissue specificity than the TSSs overlapping non-ubi-PLSs (Figure 3D; Wilcoxon rank-sum test *P*-value < 2.2 × 10^−16^; median = 0.81 and 0.96, respectively). A small number of TSSs (*n* = 2846) overlapping non-ubi-PLSs but belonged to genes that had other TSSs overlapping ubi-PLSs; these TSSs were significantly less tissue-specific than the remaining 22 208 TSSs overlapping non-ubi-PLSs (Figure 3D; Wilcoxon rank-sum test *P*-value < 2.2 × 10^−16^; median = 0.93 and 0.96, respectively). Thus, genes and TSSs associated with ubi-PLSs are broadly expressed across many biosamples.

### ubi-PLSs contain multiple TSSs and are depleted of TATA-box and narrow-peak promoters

While most human promoters have high CpG content but are depleted in TATA boxes, a subset of promoters contain a TATA box ∼30 nt upstream of the TSS. These two classes of promoters exhibit ubiquitous and tissue-specific expression, respectively, with expressed TSSs showing distinct sequencing-read profiles (called promoter shapes) (40,41). As shown above, ubi-PLSs mostly correspond to high-CG promoters with ubiquitous expression. We further found that most ubi-PLSs contained multiple GENCODE TSSs while most non-ubi-PLSs contained only one TSS (Figure 4A; Wilcoxon rank-sum test *P*-value < 2.2 × 10^−16^). Thus, we proceeded to analyze their promoter shapes.

Using RAMPAGE data in each of 115 biosamples, we classified promoters into three categories using a previous definition (41) (Supplementary Figure S5A; see Methods): narrow peaks (a narrow RAMPAGE peak with a single summit that contains most of the reads), broad peaks (a broad RAMPAGE peak with a single summit that contains most of the reads), and dispersed peaks (RAMPAGE peaks without a single summit that contains most of the reads). For example, the *SNRPD1* gene had multiple TSSs overlapping a ubi-PLS, and it contained a dispersed peak that was transcribed in both K562 and GM12878 cells (Supplementary Figure S5B). In sharp contrast, the *CALB1* gene contained one TSS in a non-ubi-PLS showing a narrow peak shape that was transcribed in K562 cells but not in GM12878 cells (Supplementary Figure S5C). In comparison with non-ubi-PLSs, ubi-PLSs were moderately but significantly enriched in dispersed-peak promoters while depleted in narrow-peak promoters in most of the 115 biosamples (Figure 4B).

The vast majority (92.71%) of ubi-PLSs overlapped CpG islands, while only half of non-ubi-PLSs overlapped CpG islands (Figure 4C). In contrast, the percentage of non-ubi-PLSs that had a TATA-box was twice that of ubi-PLSs (7.94% versus 3.03%; Figure 4C). The TATA-box preference was well-matched with promoter peak shape, with TATA promoters corresponding to narrow peaks (Figure 4C). When ubi-PLSs and non-ubi-PLSs were divided into three groups according to their promoter shapes, narrow peaks showed a prominent TATA-box at the –30 nt position while broad and dispersed peaks were depleted of any sequence motif at this position; this pattern was observed for both ubi-PLSs and non-ubi-PLSs (Figure 4D). Nevertheless, the narrow peaks in non-ubi-PLSs showed a stronger TATA motif than the narrow peaks in ubi-PLSs, while each promoter shape in ubi-PLSs were more enriched in C and G nucleotides than the corresponding promoter shape in non-ubi-PLSs (Figure 4D). Our results are consistent with the previously reported enrichment of high-CG promoters in dispersed peaks and TATA promoters in narrow peaks (40,41). In summary, ubi-PLSs and non-ubi-PLSs have distinct promoter features—ubi-PLSs tend to have multiple TSSs and are enriched in dispersed peaks while depleted of narrow peaks, and most ubi-PLSs overlap CpG islands and are unlikely to have a TATA-box.

### ubi-PLSs are enriched in the motifs of ubiquitously expressed transcription factors and the promoters preferentially responsive to EMSY and MLL3

We scanned the two sets of cCRE-PLSs against the transcription factor motifs in the JASPAR database (see Materials and Methods) and found that ubi-PLSs had significantly more motif sites than non-ubi-PLSs; collectively, these motif sites covered more base pairs in ubi-PLSs than in non-ubi-PLSs (Figure 5A). Two different sets of transcription factors corresponded to these motif sites, with the ETV and ELK families among 38 transcription factors showing the strongest enrichments for ubi-PLSs and the HNF, GATA, and AP-1 (FOS:JUN dimer) families among 119 transcription factors showing the strongest enrichments for non-ubi-PLSs (Figure 5B, Supplementary Table S4). Some of these enriched transcription factors were further supported by enrichments in binding signals using available ChIP-seq data in four human cell lines (HepG2, H1, K562 and GM12878; Supplementary Figure S6). The ChIP-seq signals were higher at ubi-PLSs than at non-ubi-PLSs for all transcription factors, regardless of their preferences for one set of cCRE-PLSs over the other (Supplementary Figure S7). The transcription factors with motif enrichment in ubi-PLSs showed ubiquitous expression profiles computed using the aforementioned RNA-seq data in 103 ENCODE biosamples (see Materials and Methods), while the transcription factors with motif enrichment in non-ubi-PLSs showed highly tissue-specific expression profiles (Figure 5C). Moreover, the transcription factors with motif enrichment in ubi-PLSs were expressed at higher levels (measured by RNA-seq in the same 16 samples as in Figure 3A) than the transcription factors with motif enrichment in non-ubi-PLSs (Supplementary Figure S8). In summary, ubi-PLSs are bound by more transcription factors and are bound more strongly than non-ubi-PLSs, and the transcription factors that regulate ubi-PLSs are more widely expressed across cell types than the transcription factors that regulate non-ubi-PLSs. These findings are highly consistent with the expression profiles of the two sets of cCRE-PLSs as described in previous sections.

We further assessed the promoter activities of the two sets of cCRE-PLSs using the STAP-seq data from a high-throughput promoter activity assay (30). The STAP-seq data were available for six transcriptional cofactors with distinct preferences for promoter types—MED15, P65, BRD4, and EP300, which prefer TATA-box promoters, and EMSY and MLL3, which prefer CpG-island promoters (30). For all six cofactors, the candidate core promoters that overlapped ubi-PLSs had significantly higher STAP-seq signals than those that overlapped non-ubi-PLSs, which were further higher than those that did not overlap either group of cCRE-PLSs (Figure 5D; Wilcoxon rank-sum test *P*-values < 4.4 × 10^−6^). This result is consistent with our earlier results on the higher expression levels for genes and TSSs that overlapped ubi-PLSs across all biosamples analyzed (Figure 3). The promoter activity profiles for the six cofactors clustered into two groups when only the candidate core promoters that overlapped cCREs were used (Figure 5E), in the same way as reported previously with all candidate core promoters (30). The two cofactors that were known to prefer CpG-island promoters over TATA-box promoters—EMSY and MLL3—exhibited the highest ratios of median signals among the candidate core promoters that overlapped ubi-PLSs over the median signals among the candidate core promoters that overlapped non-ubi-PLSs (Figure 5F). This result is also consistent with our earlier results on the enrichment of ubi-PLSs in CpG-island promoters (Figure 4). Thus, ubi-PLSs have higher promoter activities than non-ubi-PLSs, and they prefer different transcriptional cofactors.

### ubi-PLSs are highly conserved between human and mouse

To explore the evolutionary conservation of ubi-PLSs between human and mouse, we defined mouse ubi-PLSs in the same way as human ubi-PLSs (see Materials and Methods). Mouse rDHSs with high DNase signal in at least 90 biosamples (out of a total of 94 biosamples with DNase-seq data) were defined as ubi-rDHSs (Figure 6A; *n* = 13 247). Similar to the human ubi-rDHSs, the majority of mouse ubi-rDHSs were TSS-proximal (60% were cCRE-PLS and 22% were cCRE-pELS; Figure 6B); in total, there were 7907 mouse ubi-PLSs (Supplementary Table S2D). These ubi-PLSs overlapped 12 745 GENCODE M18 TSSs, which belonged to 8051 genes (Supplementary Table S2E, F).

The vast majority of ubi-PLSs and non-ubi-PLSs in both species could be lifted over to the reciprocal genome (see Materials and Methods), with a slightly higher mapping rate for ubi-PLSs (87% from human to mouse and 91% from mouse to human) than for non-ubi-PLSs (81% from human to mouse and 90% from mouse to human; Figure 6C). Most of the syntenic regions of ubi-PLSs in the other genome were still ubi-PLSs (63% of the human ubi-PLSs and 71% of the mouse ubi-PLSs). In sharp contrast, much lower percentages of the syntenic regions of non-ubi-PLSs remained non-ubi-PLSs in the other species (25% of human non-ubi-PLSs and 39% of mouse non-ubi-PLSs); they became cCRE-pELSs or were no longer rDHSs (Figure 6C). Thus, ubi-PLSs are much more evolutionarily conserved than non-ubi-PLSs in terms of synteny and ubiquity of chromatin accessibility.

Like human ubi-PLSs (Figure 5A), the syntenic regions of human ubi-PLS in mouse had significantly more motif sites than the syntenic regions of human non-ubi-PLS in mouse, and collectively, these motif sites covered more base pairs in the syntenic regions of ubi-PLSs than non-ubi-PLSs (Figure 6D).

We further examined the sequence conservation of the ±2 kb region centered on the two sets of cCRE-PLSs using two metrics for evolutionary conservation, phastCons and phyloP (42–44). The two sets show similar levels of conservation judged by phastCons, but judged by phyloP, which has a higher resolution than phastCons, ubi-PLSs are less conserved than non-ubi-PLSs in the small window immediately upstream of the cCRE-PLS center (Figure 6E). This small window corresponds to the core promoter where most transcription factors bind. We described above that ubi-PLSs had more transcription factor motif sites than non-ubi-PLSs (Figures 5A and 6D), so we asked whether these motif sites maintained their positions between human and mouse cCRE-PLSs. We found that the motif sites in ubi-PLSs differed more in their distances to the nearest TSS between human and mouse than the motif sites in non-ubi-PLSs (Figure 6F). Thus, turnover of transcription factor binding sites may be a cause for the lower sequence conservation in the core promoter regions in ubi-PLSs than those in non-ubi-PLSs.

### cCRE-PLSs that overlap cell-essential genes or Mendelian-disease genes are more conserved between human and mouse than those that do not

We divided human ubi-PLSs into two groups according to whether they overlapped the TSSs of 5312 genes deemed cell essential in at least one of the previous three studies (15–17), and likewise for non-ubi-PLSs. Among the cell-essential sub-set of ubi-PLSs (*n* = 4216 of 9098), 91.8% can be lifted over to the mouse, while only 83.7% of the remaining ubi-PLSs can be lifted over (Fisher's exact test *P*-value = 1.67 × 10^−32^). Similarly, the non-ubi-PLSs that overlap cell-essential genes (*n* = 3982 of 25 705) have a significantly higher percentage of being lifted over to the mouse than the remaining non-ubi-PLSs (85.9% versus 79.6%, Fisher's exact test *P*-value = 7.39 × 10^−22^). Furthermore, the cell-essential subsets show higher phyloP scores than the respective non-cell-essential subsets (Figure 7A). These results are consistent with the lower dn/ds ratios (nonsynonymous vs. synonymous mutations) in the coding regions of genes that are more cell essential (15).

We also considered a set of 2759 genes implicated in Mendelian diseases (31). These Mendelian-disease genes overlap significantly, albeit moderately, with the above 5312 cell-essential genes (941 Mendelian-disease genes are cell essential, Fisher's exact test *P*-value = 6.1 × 10^−14^). There is a 1.75-fold enrichment of ubi-PLSs that overlap the TSSs of Mendelian-disease genes over non-ubi-PLSs (20.6% versus 11.8%, *P*-value = 3.51 × 10^−35^). The ubi-PLSs that overlap Mendelian-disease genes have a higher chance of being lifted over to the mouse (92.2% versus 86.7%, *P*-value = 1.52 × 10^−6^). We observed the same trend for non-ubi-PLSs, with an even greater effect (91.1% versus 79.1%, *P*-value = 1.55 × 10^−64^). The 3043 non-ubi-PLSs that overlap Mendelian-disease genes have much higher phyloP scores than the remaining 22 662 non-ubi-PLSs, which show similar levels of phyloP scores to ubi-PLSs regardless of whether they overlap Mendelian-disease genes (Figure 7B). These results further suggest that ubi-PLSs and non-ubi-PLSs are regulated differently.

### ubi-PLSs show high variation at the center but low variation in flanking regions in human populations

To examine the evolutionary conservation in the time scale of human evolution, we computed the nucleotide diversity (π; see Materials and Methods) at cCRE-PLSs and their surrounding genomic regions (2 kb upstream and 2 kb downstream) using the whole-genome sequencing data of ∼186 000 individuals from the TOPMed project (32). π is a metric of sequence variation in a population, reflecting the elimination of deleterious alleles by natural selection thus it is indicative of functional constraint in recent evolution. ubi-PLSs have significantly lower π than non-ubi-PLSs at both upstream and downstream regions, but substantially higher π at the ubi-PLSs (with the summit of the π profile shifted slightly upstream), even higher than genome-wide average (Figure 6G, Supplementary Figure S9A). This high π value at ubi-PLSs correspond to the aforementioned dip in the phyloP profile (Figure 6E), likely reflecting the rapid turnovers of TF binding sites in ubi-PLSs (Figure 6F).

The subset of ubi-PLSs that overlap the TSSs of cell-essential genes show lower π than the remaining ubi-PLSs (Figure 7C, Supplementary Figure S9B). Likewise, the subset of non-ubi-PLSs that overlap the TSSs of cell-essential genes show substantially lower π than the remaining ubi-PLSs (Figure 7C, Supplementary Figure S9B). These results are consistent with our results on the cross-species phyloP conservation score (Figure 7A).

The subset of ubi-PLSs that overlap the TSSs of Mendelian-disease genes show significantly higher π than the remaining ubi-PLSs (Figure 7D, Supplementary Figure S9C), although the phyloP scores at these two subsets of ubi-PLSs do not differ (Figure 7B). Intriguingly, the non-ubi-PLSs that do not overlap Mendelian-disease genes show the highest π in the ±2 kb region centered on cCRE-PLSs among the four subsets (Figure 7D, Supplementary Figure S9C), although their phyloP scores are comparable with those of ubi-PLSs (Figure 7B). These quantitative differences between our results using π and phyloP indicate that the non-ubi-PLSs that overlap Mendelian-disease genes are under more evolutionary constraint than other cCRE-PLSs in the time scale of vertebrate evolution while the non-ubi-PLSs that do not overlap Mendelian-disease genes are under less evolutionary constraint than other cCRE-PLSs in the time scale of human evolution.

To investigate whether the high π for the ubi-PLSs associated with Mendelian-disease genes was due to the depletion of cell-essential genes in this sub-group, we further stratified our data by both Mendelian-disease and cell-essential classifications. Compared with non-ubi-PLSs, ubi-PLSs are enriched in both Mendelian-disease, cell-essential and non-Mendelian-disease, cell-essential sub-groups (Supplementary Figure S9D), and the Mendelian-disease cell-essential sub-group shows significantly higher π than other sub-groups (Supplementary Figure S9E), confirming that high DNA diversity is an intrinsic feature of ubi-PLSs.

### ubi-PLSs and non-ubi-PLSs are differentially associated with Mendelian diseases

Compared with non-ubi-PLSs, ubi-PLSs are highly enriched in the genes associated with two related sets of Mendelian diseases that impact all cell types (Supplementary Figure S9F,H): mitochondrial complex deficiency and oxidative phosphorylation deficiency, which impedes the main function of mitochondria—producing energy to fuel all cells in the body, especially the nervous system, heart, and skeletal muscles. There are 78 genes implicated in these diseases, 31 have only ubi-PLSs, 3 have only non-ubi-PLSs, and 22 have both types of cCRE-PLSs (Fisher's exact test *P*-value = 5.84 × 10^−21^, Supplementary Table S5).

In contrast, non-ubi-PLSs are highly enriched in the genes associated with Mendelian diseases that impact subsets of cell types, for example, deafness and myotrophy (Supplementary Figure S9G,H). There are 79 genes implicated in deafness, 0 have only ubi-PLSs, 59 have only non-ubi-PLSs, and 10 have both types of cCRE-PLSs (Fisher's exact test *P*-value = 2.65 × 10^−4^, Supplementary Table S5). The non-ubi-PLSs associated with deafness genes tend to have open chromatin in fibroblasts, endothelial cells in blood vessels, and related cell types, while closed chromatin in other cell types such as immune cells. There are 70 genes implicated in myopathy; 3 have only ubi-PLSs, 49 have only non-ubi-PLSs and 9 have both types of cCRE-PLSs (Fisher's exact test *P*-value = 1.23 × 10^−2^, Supplementary Table S5). The non-ubi-PLSs associated with myopathy genes tend to have open chromatin in muscle cells, especially cardiac muscles in the heart.

## DISCUSSION

We systematically analyzed 9098 human promoters and 7907 mouse promoters that had open chromatin in >95% of the human and mouse biosamples tested. Compared with non-ubi-PLSs, ubi-PLSs have seven striking genomic and epigenomic features in addition to having ubiquitously open chromatin. First, they have high CG content and lack a TATA box (Figures 2A, 4C). Second, they mostly belong to housekeeping genes, being highly enriched in GO categories of metabolism and biosynthesis and highly depleted in GO categories of signal transduction and stimulus detection (Figure 1E, F). Accordingly, they are highly enriched in cell-essential genes (Figure 1G, H). Third, these ubi-PLSs show high levels of open chromatin (Figure 2B), active histone modifications, well-positioned flanking nucleosomes (Supplementary Figure S2), and high levels of chromatin interactions (Supplementary Figure S3). Fourth, they are highly expressed (at both the gene and TSS levels) across cell and tissue types (Figure 3, Supplementary Figure S4). Fifth, transcription tends to fire from multiple positions in ubi-PLSs, hence ubi-PLSs are enriched in the dispersed-peak promoter shape and depleted in the sharp-peak promoter shape (Figure 4). Sixth, they are likely regulated by a distinct set of transcription factors (based on motif and ChIP-seq peak enrichments), which also tend to be ubiquitously expressed (Figure 5, Supplementary Figure S6), and they are co-regulated by transcriptional cofactors EMSY and MLL3, which prefer CpG-island promoters (Figure 5). Seventh, they are highly conserved between human and mouse at the synteny level, and most of them are ubi-PLSs in both species; however, they are not as conserved at the sequence level, with a high turnover of transcription factor motif sites (Figure 6). Furthermore, they show low variation among fully sequenced human genomes in surrounding regions but high variation at the center (Figure 6G, Supplementary Figure S9A–E).

Collectively, these genomic and epigenomic features point to a highly consistent model (Figure 7E) for the transcriptional regulation of roughly nine thousand genes that are expressed in most cell types. With highly G/C rich sequences, the DNA of ubi-PLSs would be bound by nucleosomes *in vitro* (20) (Figure 2C), indicating that it is not the DNA sequences of the ubi-PLSs that have the inherent ability to keep them open chromatin across most cell types. Rather, it is more likely that the occupancy by transcription factors, especially the transcription factors that are expressed in most cell types, competes with histone proteins for accessing the DNA and keeps the ubi-PLSs in the open chromatin state. Indeed, the high ChIP-seq signals of most transcription factors at ubi-PLSs (Supplementary Figure S7) support their high occupancy on the ubi-PLSs.

Typically, multiple transcription factors bind to a promoter and regulate its transcription, and two broad classes of models have been proposed for the mechanisms of gene regulation: the regulatory grammar model (45,46) and the flexible billboard model (47,48). The regulatory grammar model states that a specific syntax of transcription factor binding (e.g. the relative orientation and distance between neighboring sites) is required for the co-regulation to occur, while the flexible billboard model states that the composition of the bound transcription factors, but not so much their relative orientations and positions, determines the co-regulation. We found that between human and mouse, most syntenic regions of ubi-PLSs in one species were also ubi-PLSs in the other species, although their DNA sequences were less conserved, with movements of transcription factor motif sites (Figure 6C–F). Similarly, in human populations, the regions surrounding ubi-PLSs show decreased variation although the ubi-PLSs themselves show increased variation (Figure 6G). Our results suggest that ubi-PLSs are more likely to adopt the flexible billboard model—although the transcription factor sites can change their positions between human and mouse, the promoters remain functional as ubi-PLSs in both species.

Our findings of ubi-PLSs are highly consistent with earlier work based on transcriptome data (40,41). An earlier study used CAGE data to define different promoter shapes and then analyzed the sequence features for the different promoter shapes, showing that broad-peak promoters were high in CG and lacked a TATA-box (40). We started with chromatin accessibility data and defined a set of promoters that had open chromatin in most biosamples, and then we showed that ubi-PLSs were enriched in broad-peak, high CG promoters. The earlier study reported that the high CG promoters were more rapidly evolving in mammals than TATA-containing promoters. In agreement with these earlier findings, we found lower sequence conservation in the regions immediately upstream the center positions of ubi-PLSs with respect to the direction of transcription than in the corresponding regions of non-ubi-PLSs between human and mouse (Figure 6E). These regions around ubi-PLSs also show increased human variation (Figure 6G). Thus, our results added chromatin and epigenetic data to the previous knowledge of promoter types defined using transcriptome data, completing our understanding of how promoters are regulated.

High-throughput techniques such as massively parallel reporter assays (MPRA) are increasingly used to dissect the regulatory mechanisms of regulatory elements (49–52). Some studies insert transcription factor motif sites into synthetic constructs, while other studies eliminate existing motif sites in genomic sequences, and then the impact of these changes is measured by a reporter. One recurrent finding is that the degree of transcription factor occupancy (often represented by the number of motif sites) is one of the best predictors for the reporter expression (49–52). Our finding of larger numbers of motif sites in ubi-PLSs and their higher expression levels than non-ubi-PLSs agrees with the earlier findings of MPRA studies (50). Furthermore, we found a large enrichment of ubi-PLSs in bidirectional promoters, which agrees with the earlier finding that bidirectional promoters were more active than unidirectional promoters in driving reporter expression (50). We found GABPA, E2F2/3, and YY1/2 to be among the 38 transcription factors whose motifs were enriched in ubi-PLSs, and these motifs were found earlier to be enriched in bidirectional promoters, especially GABPA (53). We also found that ubi-PLSs were enriched in the motifs of ubiquitously expressed transcription factors, including the ETV and ELK families, and these motifs were also found to be enriched in the promoters of ubiquitously expressed genes (54).

It is interesting that ubi-PLSs are more enriched than non-ubi-PLSs in Mendelian-disease genes in general and the Mendelian diseases preferentially associated with ubi-PLSs affect ubiquitous cellular functions such as the oxidative phosphorylation function carried out by the mitochondrial complexes (Supplementary Figure S9F–H). Moreover, it is intriguing that the ubi-PLSs associated with Mendelian-disease genes show increased nucleotide diversity than the non-Mendelian-disease subsets (Supplementary Figure S9A–E). We hypothesize that the distinct regulatory mechanisms of these two sets of promoters (Figure 7E) may be connected with their differential disease susceptibilities.

In summary, we have performed extensive analyses on roughly nine thousand human promoters that are open chromatin in more than 95% of the biosamples. Our analyses showed that these promoters are likely regulated by a common mechanism at the center of which is a small set of widely expressed transcription factors. These promoters are mostly syntenic between human and mouse and are conserved in their ubiquitously expressed promoter function. They are essential for maintaining the high-level transcription of a set of genes required for the normal function of most cell types.

## DATA AVAILABILITY

All processed datasets are available. cCREs and rDHSs are available at the ENCODE portal (www.encodeproject.org) with the accessions listed in Supplementary Table S1. ubi-rDHSs are listed in Supplementary Table S2. ChIA-PET datasets were downloaded from GEO under the accession GSE72816, STAP-seq datasets were downloaded from GEO under GSE126221, and *in vitro* MNase-seq data was downloaded from GEO under the accession GSE25133. ENCODE RNA-seq, RAMPAGE, ChIP-seq and MNase-seq experiments are available at the ENCODE portal with accessions listed in Supplementary Table S1.

## Supplementary Material

gkab345_Supplemental_Files
