# Supplementary material for: Genetic and epigenetic features of promoters with ubiquitous chromatin accessibility support ubiquitous transcription of cell-essential genes
Source: Nucleic Acids Res. 2021 May 12;49(10):5705–25. doi: 10.1093/nar/gkab345 (PMC8191798; doi:10.1093/nar/gkab345)
Supplement: gkab345_Supplemental_Files [file gkab345_supplemental_files.zip › Fan et al. Supplementary Figures.pdf]

Supplementary Figures

Genetic and Epigenetic Features of Promoters with Ubiquitous Chromatin Accessibility Support Ubiquitous Transcription of Cell-essential Genes

Kaili Fan[1], Jill E. Moore[1], Xiao-ou Zhang[1], and Zhiping Weng[1, *]

[1]Program in Bioinformatics and Integrative Biology, UMass Medical School, Worcester, MA, USA.

* Correspondence should be addressed to ZW (zhiping.weng@umassmed.edu).

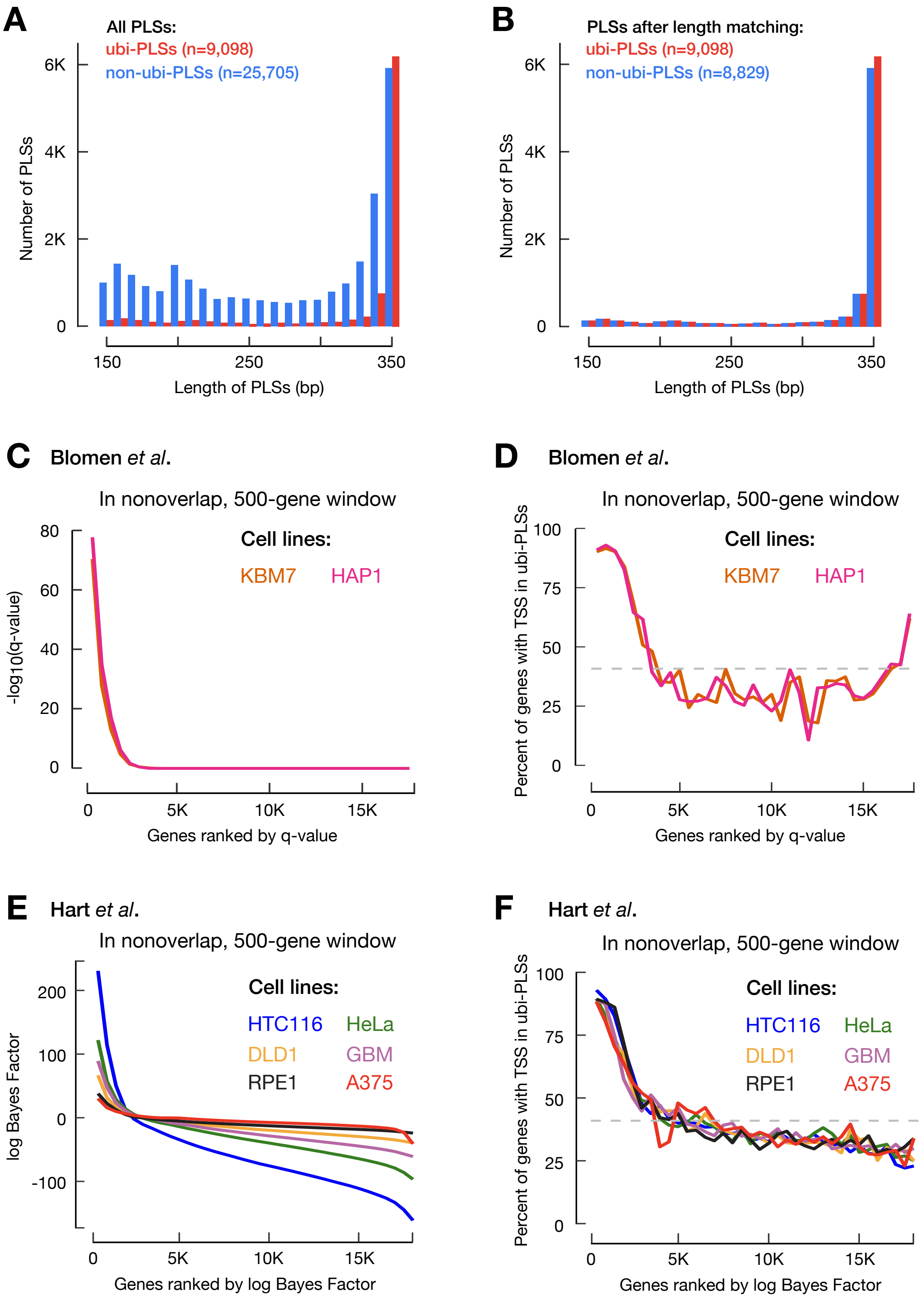

Supplementary Figure S1. ubi-PLSs are the TSSs of cell-essential genes

A. Length distributions of all ubi-PLSs (red) and all non-ubi-PLSs (blue).

B. Length distributions of all ubi-PLSs (red) and a subset of non-ubi-PLSs randomly selected to match the length distribution of ubi-PLSs (blue).

C-F. Similar to **Figure 1H**, but with cell-essential genes from the other two studies (Blomen *et al.* in **C** and **D**, Hart *et al.* in **E** and **F**).

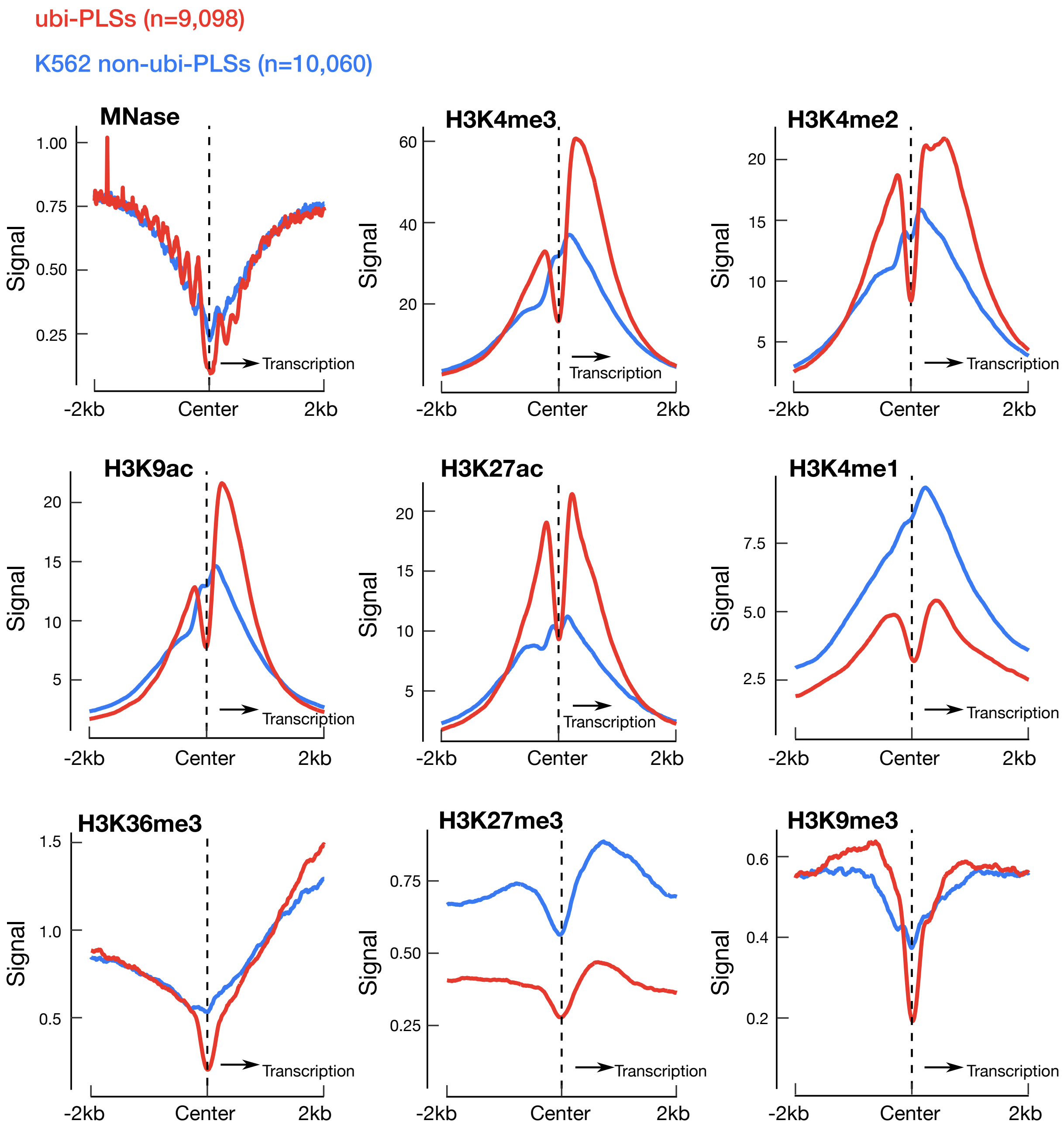

Supplementary Figure S2. ubi-PLSs have higher signals of active histone marks and lower signals of repressive histone marks than non-ubi-PLSs.

Aggregation plots depict the signal profiles of histone marks and MNase in genomic regions centered on ubi-PLSs (red) versus non-ubi-PLSs (blue) in K562 cells. Active histone marks include H3K4me3, H3K4me2, H3K27ac, H3K9ac, H3K4me1, and H3K36me3, while repressive histone marks include H3K27me3 and H3K9me3. All histone marks signals were obtained from ChIP-seq data, quantified by fold change over control. Arrows in the figure represent the transcriptional direction of the associated genes.

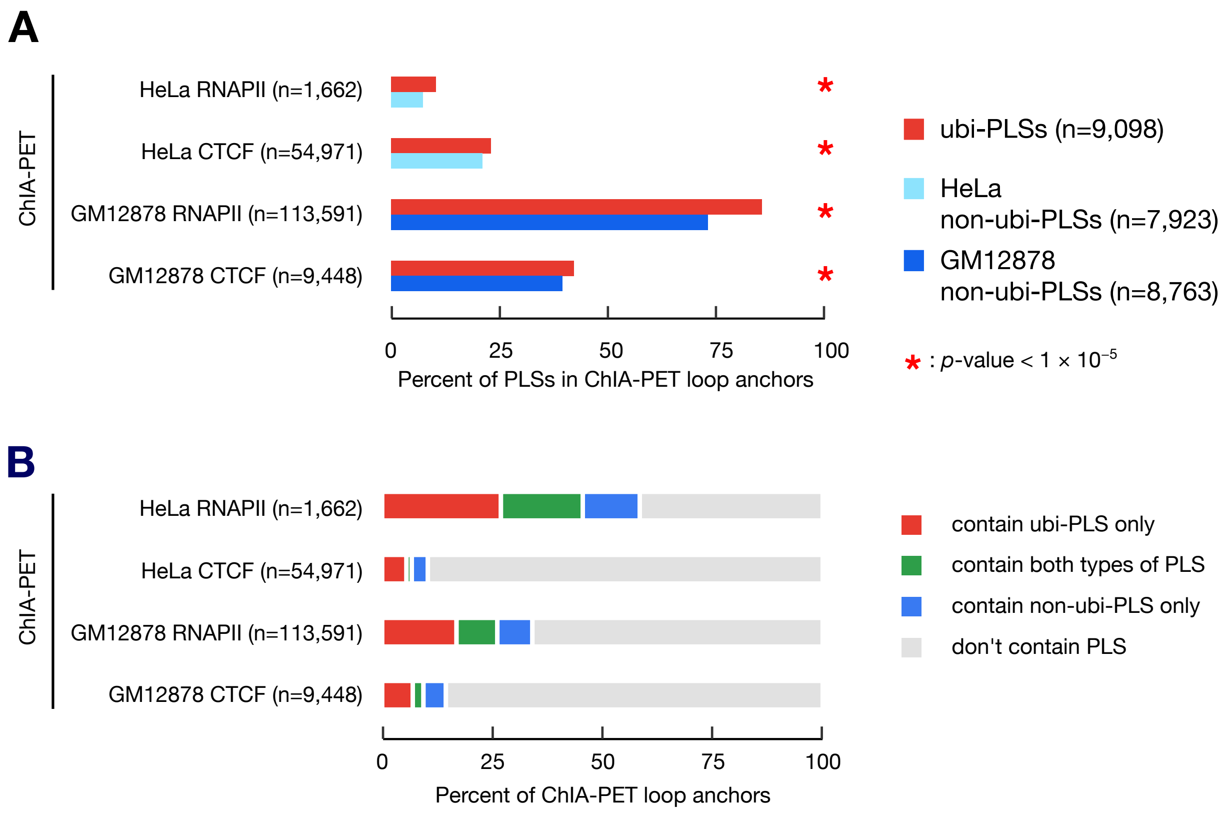

Supplementary Figure S3. ubi-PLSs are enriched in ChIA-PET loop anchors.
A. Higher percentages of ubi-PLSs are located in the loop anchors defined by ChIA-PET than the non-ubi-PLSs in the same cell type as the ChIA-PET data. Four ChIA-PET datasets were used: RNA Pol II (RNAPII) in HeLa cells, CTCF in HeLa cells, RNAPII in GM12878 cells, and CTCF in GM12878 cells. ubi-PLSs are shown in red, while non-ubi-PLSs are shown in different shades of blue. All *p*-values were computed with Fisher's exact tests.
B. A bar plot shows that higher percentages of ChIA-PET loop anchors contain ubi-PLSs than non-ubi-PLSs. For each ChIA-PET dataset, the plot shows loop anchors that only contain ubi-PLSs (red), contain both ubi-PLSs and non-ubi-PLSs (green), only contain non-ubi-PLSs (blue), or do not contain any cCRE-PLSs defined in that cell type (gray).

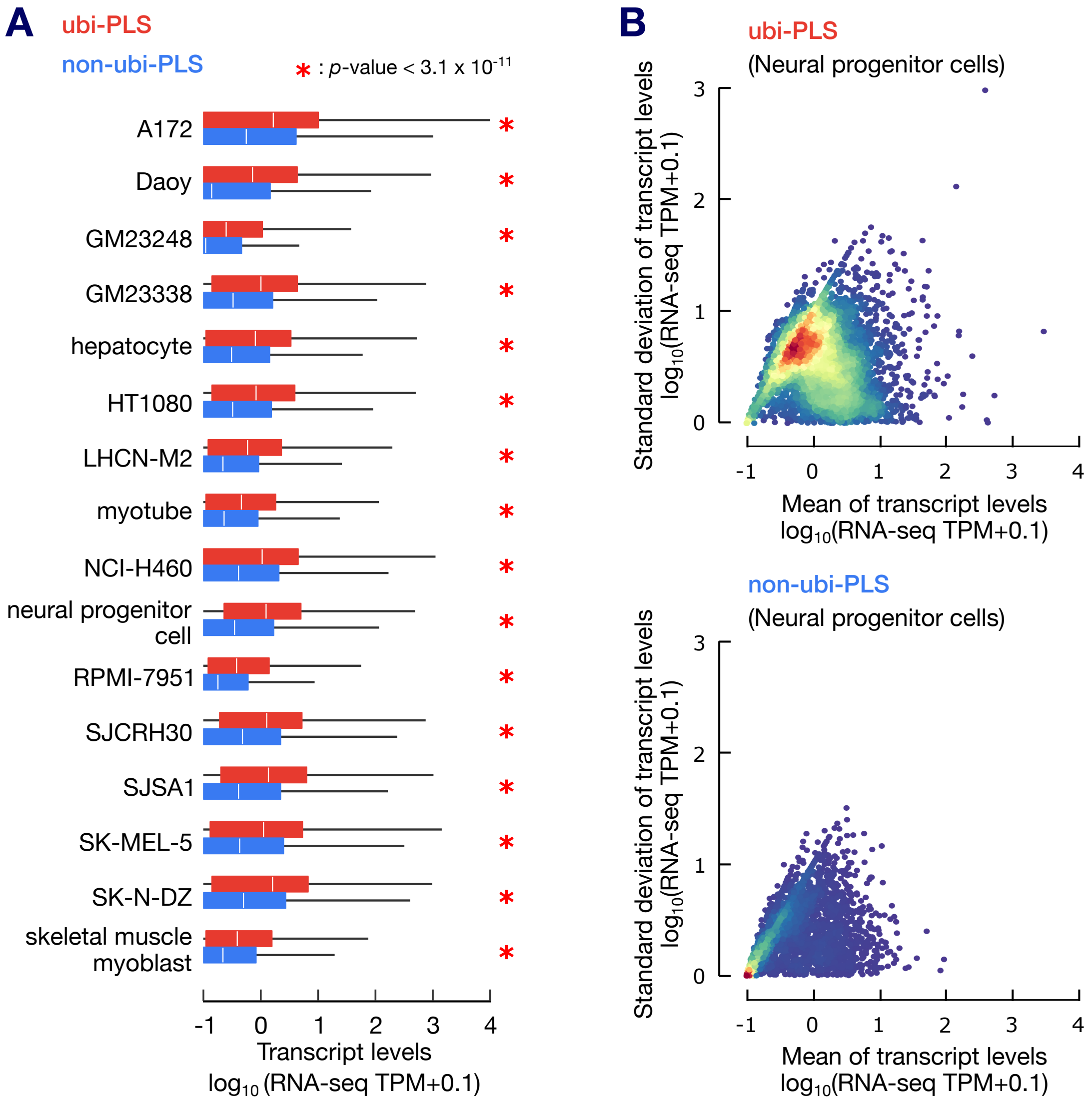

Supplementary Figure S4. ubi-PLS transcripts have higher expression levels than non-ubi-PLS transcripts.

A. Transcripts whose TSSs overlapping ubi-PLSs (red) are significantly more highly expressed than transcripts in the same gene but whose TSSs overlap the non-ubi-PLSs defined in the same biosample (blue). Expression levels were obtained from RNA-seq data, quantified by TPM (with a pseudocount of 0.1 added to each transcript), and plotted in the log scale. All *p*-values were computed with Wilcoxon rank-sum tests.

B. Transcripts with TSSs overlapping the same ubi-PLSs (top) or the same non-ubi-PLSs (bottom) can be expressed at different levels. Each point denotes one PLS and the standard deviation of the expression levels of the transcripts whose TSSs overlap the PLS is plotted against the mean expression level of these transcripts. Expression levels were obtained from RNA-seq data in neural progenitor cells (similar patterns were observed for other biosamples), quantified by TPM (with a pseudocount of 0.1 added to each transcript), and plotted in the log scale.

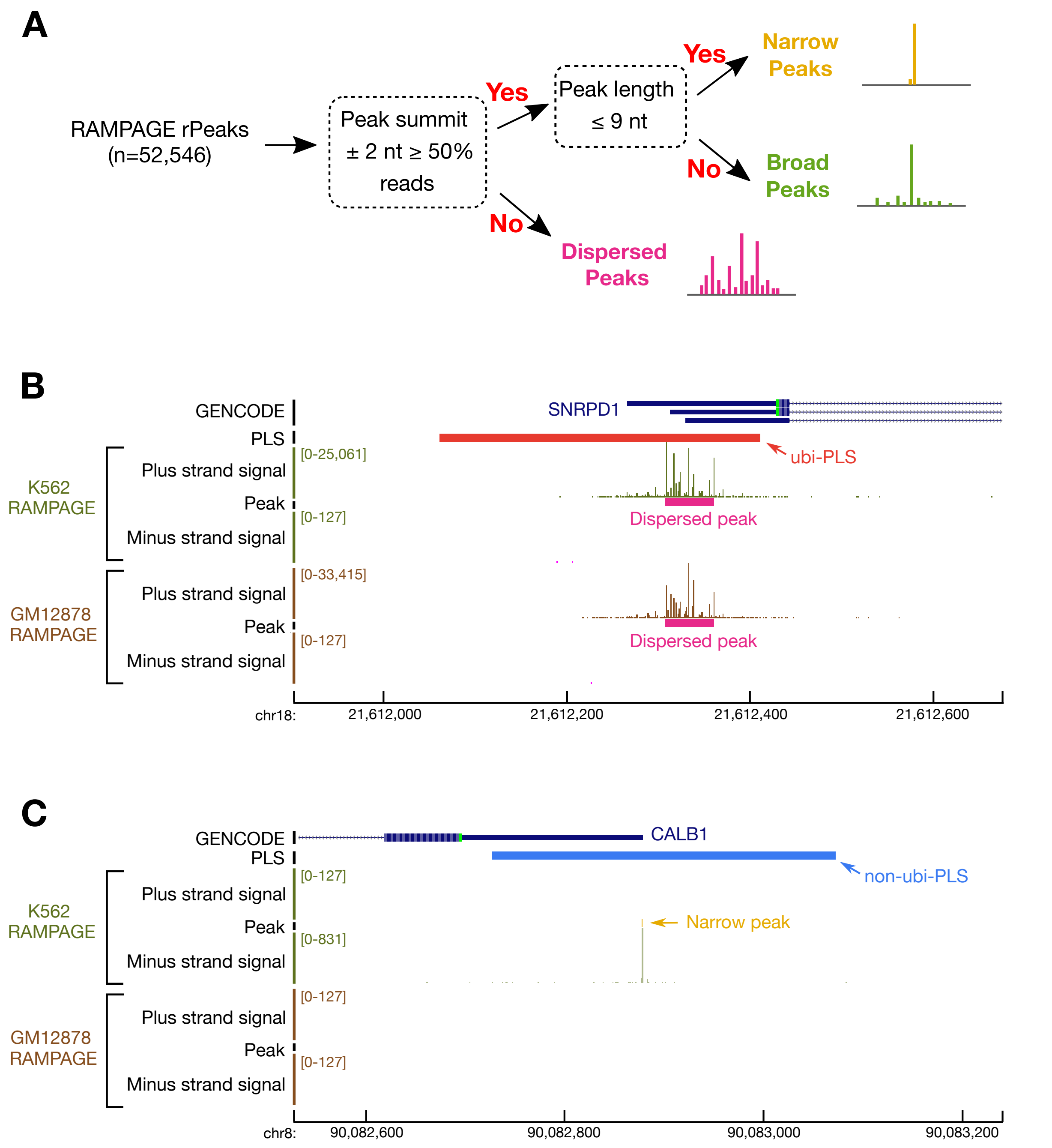

Supplementary Figure S5. Workflow for defining promoter shapes and examples of ubi-PLSs and non-ubi-PLSs with different promoter shapes.

A. Workflow for defining promoter shapes (broad peaks, narrow peaks, and dispersed peaks).

B. A ubi-PLS overlaps with a dispersed peak in K562 and GM12878 cells.

C. A non-ubi-PLS overlaps with a narrow peak in K562 cells, but this TSS is not active in GM12878 cells.

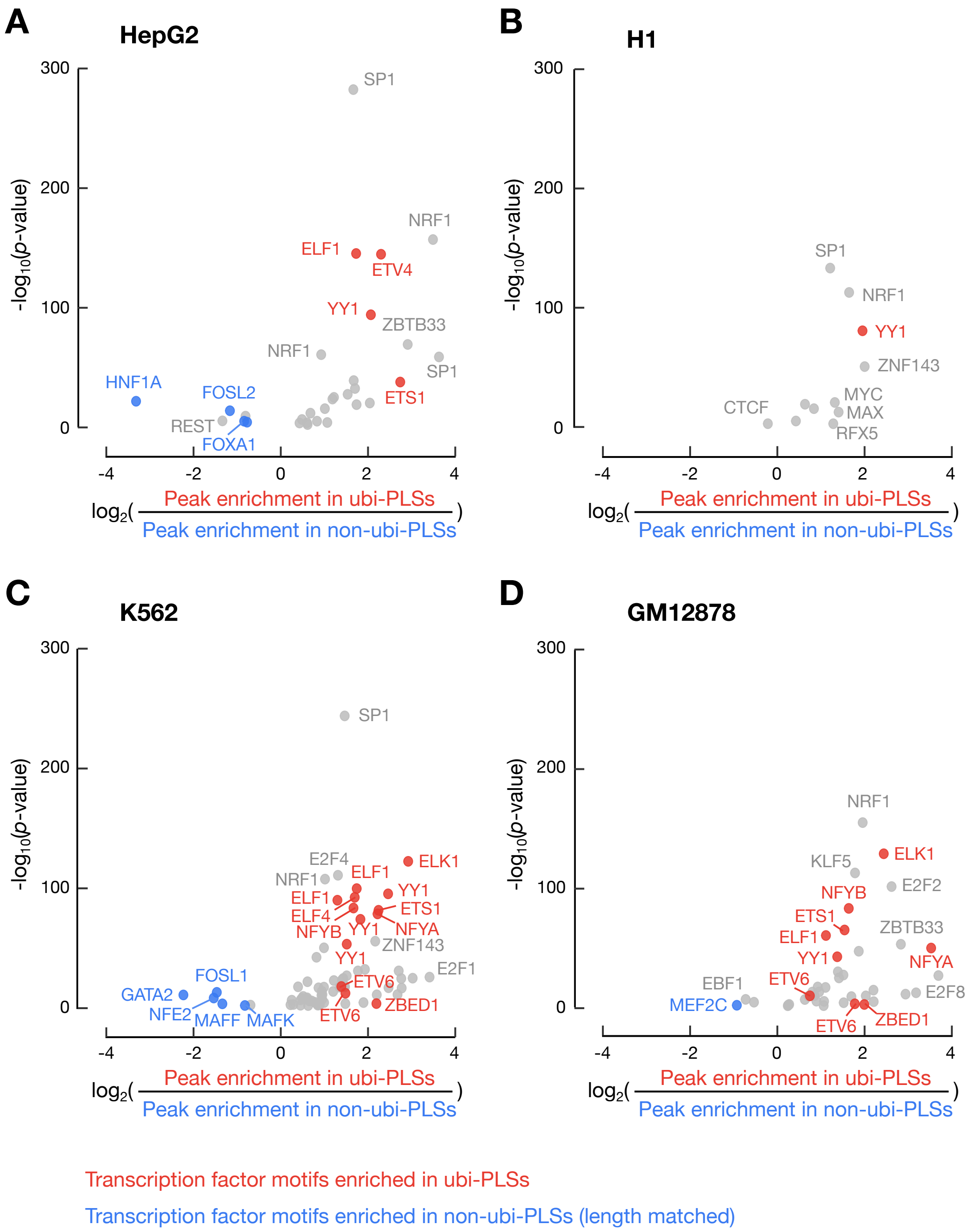

Supplementary Figure S6. Different transcription factors bind ubi-PLSs than non-ubi-PLSs.

Each dot is a transcription factor with ChIP-seq data (transcription factors with multiple ChIP-seq datasets are represented by multiple dots, e.g., SP1 in HepG2). Similar to **Figure 4B**, but the enrichment of a transcription factor is computed using their ChIP-seq peak with a motif site in HepG2 (**A**), H1 (**B**), K562 (**C**), and GM12878 (**D**) cells. As in **Figure 4B**, transcription factors whose motifs are enriched in ubi-PLSs are in red, while transcription factors whose motifs are enriched in non-ubi-PLSs are in blue. Only transcription factors with enrichment *p*-values < 0.01 are shown.

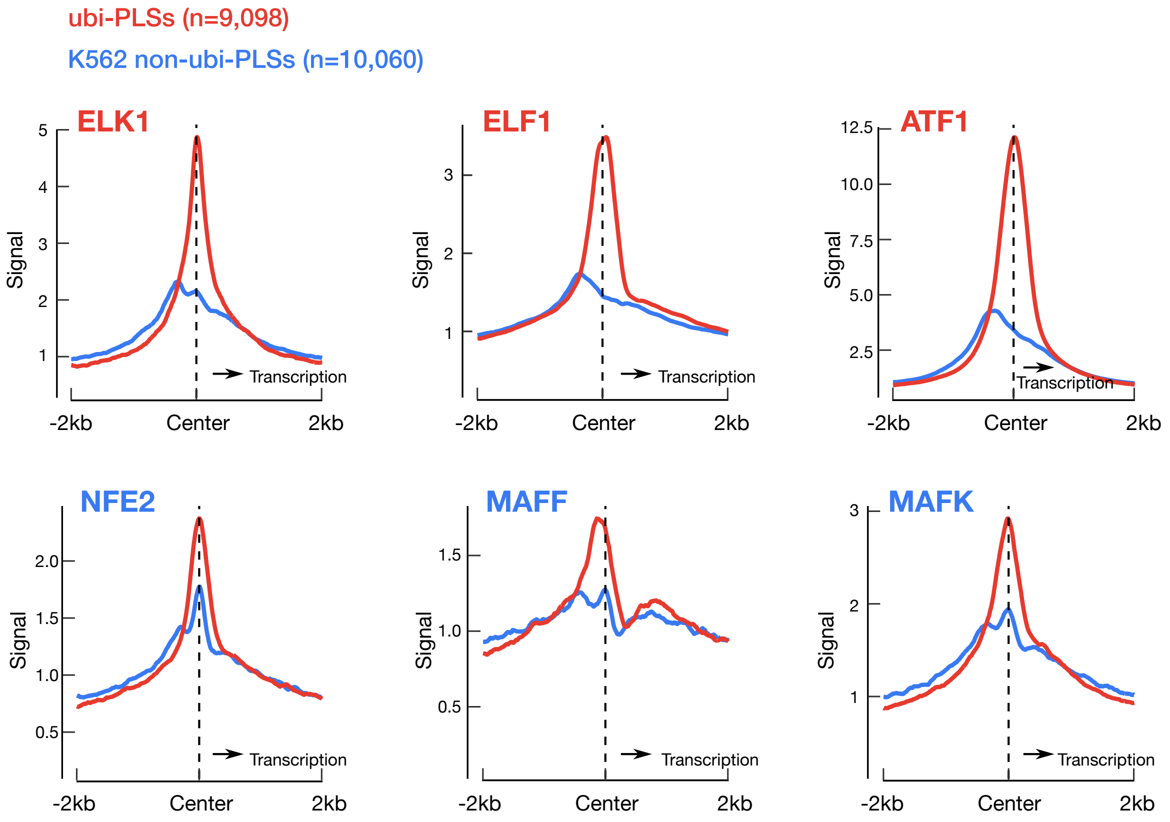

Supplementary Figure S7. Transcription factors prefer to bind ubi-PLSs rather than non-ubi-PLSs.

Aggregation plots show the ChIP-seq signal profiles for six example transcription factors in the genomic regions centered on ubi-PLSs (red) and K562 non-ubi-PLSs (blue). (top) ELK1, ELF1, and ATF1 were among the transcription factors with motifs more enriched in ubi-PLSs than in non-ubi-PLSs. (bottom) NFE2, MAFF, and MAFK were among the transcription factors with motifs more enriched in non-ubi-PLSs than in ubi-PLSs.

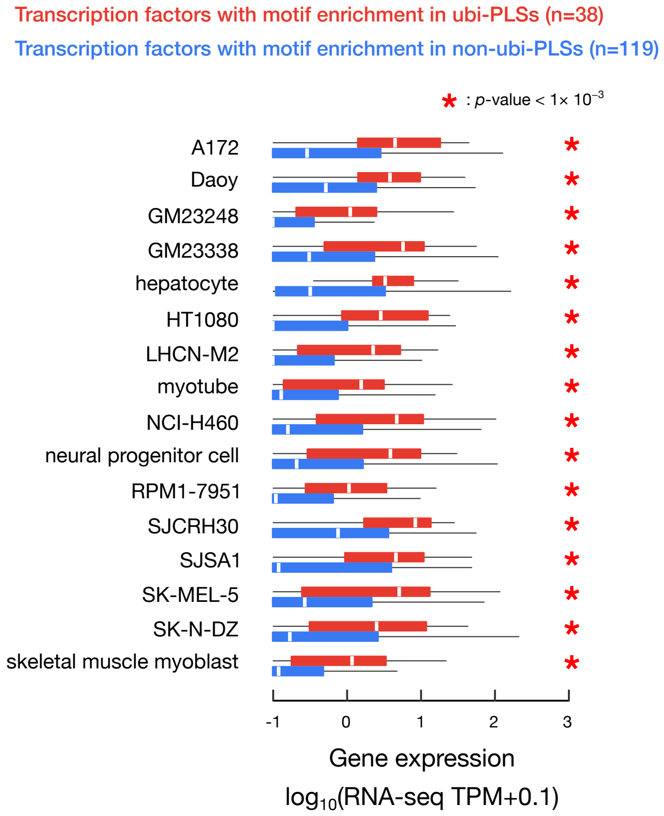

Supplementary Figure S8. Transcription factors with motif enrichment in ubi-PLSs have higher expression levels than transcription factors with motif enrichment in non-ubi-PLSs.

Boxplots show that the 38 transcription factors with motif enrichment in ubi-PLSs (red) were significantly more highly expressed than the 119 transcription factors with motif enrichment in non-ubi-PLSs (blue) in the same biosample. Expression levels were obtained from RNA-seq data, quantified in TPM (with a pseudocount of 0.1 added to each transcription factor gene), and plotted in log scale. All *p*-values were computed with Wilcoxon rank-sum tests.

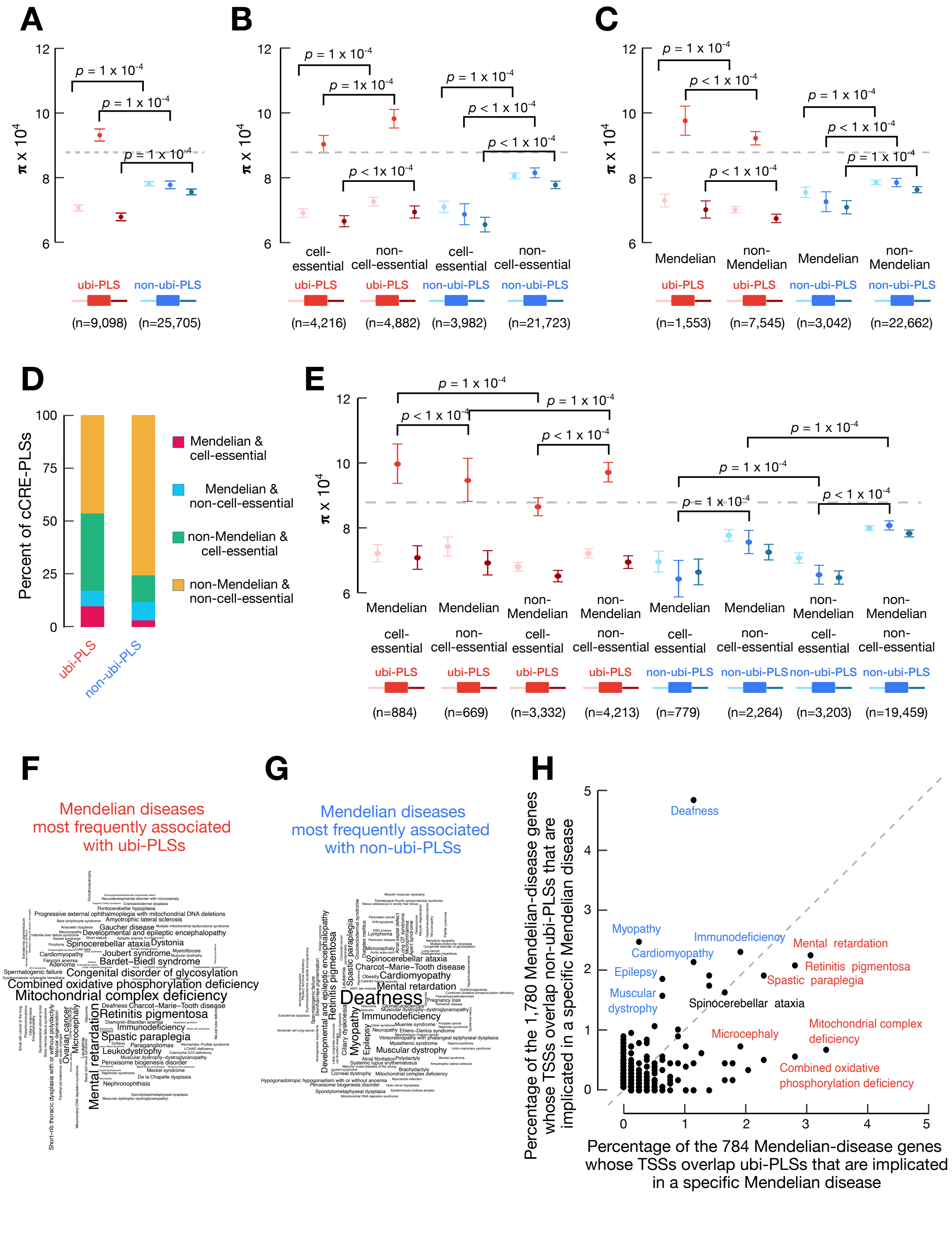

A
B
C
D
E
F
G
H
π x 10^4
p = 1 x 10^-4
p < 1 x 10^-4
ubi-PLS
non-ubi-PLS
(n=9,098)
(n=25,705)
cell-essential
non-cell-essential
(n=4,216)
(n=4,882)
(n=3,982)
(n=21,723)
Mendelian
non-Mendelian
(n=1,553)
(n=7,545)
(n=3,042)
(n=22,662)
Percent of cCRE-PLSs
Mendelian & cell-essential
Mendelian & non-cell-essential
non-Mendelian & cell-essential
non-Mendelian & non-cell-essential
(n=884)
(n=669)
(n=3,332)
(n=4,213)
(n=779)
(n=2,264)
(n=3,203)
(n=19,459)
Mendelian diseases most frequently associated with ubi-PLSs
Mitochondrial complex deficiency
Combined oxidative phosphorylation deficiency
Congenital disorder of glycosylation
Retinitis pigmentosa
Spastic paraplegia
Mental retardation
Bardet–Biedl syndrome
Joubert syndrome
Spinocerebellar ataxia
Dystonia
Developmental and epileptic encephalopathy
Gaucher disease
Progressive external ophthalmoplegia with mitochondrial DNA deletions
Amyotrophic lateral sclerosis
Pontocerebellar hypoplasia
Charcot–Marie–Tooth disease
Immunodeficiency
Leukodystrophy
Paragangliomas
Peroxisome biogenesis disorder
Microcephaly
Ovarian cancer
Deafness
Nephronophthisis
Mendelian diseases most frequently associated with non-ubi-PLSs
Deafness
Mental retardation
Cardiomyopathy
Charcot–Marie–Tooth disease
Spinocerebellar ataxia
Immunodeficiency
Muscular dystrophy
Myopathy
Epilepsy
Developmental and epileptic encephalopathy
Retinitis pigmentosa
Spastic paraplegia
Percentage of the 1,780 Mendelian-disease genes whose TSSs overlap non-ubi-PLSs that are implicated in a specific Mendelian disease
Percentage of the 784 Mendelian-disease genes whose TSSs overlap ubi-PLSs that are implicated in a specific Mendelian disease
Deafness
Myopathy
Immunodeficiency
Cardiomyopathy
Mental retardation
Epilepsy
Retinitis pigmentosa
Spastic paraplegia
Muscular dystrophy
Spinocerebellar ataxia
Microcephaly
Mitochondrial complex deficiency
Combined oxidative phosphorylation deficiency

Supplementary Figure S9. ubi-PLSs show high variation at the center but low variation in flanking regions in human populations.

A. The median and 95th-percentile confidence interval of nucleotide diversity (**π**) are shown for ubi-PLSs (in red), their 2 kb upstream regions (pink), and their 2 kb downstream regions (dark red). Similarly, the median and 95th-percentile confidence interval are shown for non-ubi-PLSs (in blue), their 2 kb upstream regions (cyan), and their 2 kb downstream regions (dark blue). Confidence intervals and *p*-values were computed by bootstrapping (see **Methods** for details).
B. Same as panel A but both ubi-PLSs and non-ubi-PLSs are stratified by association with cell-essential genes.
C. Same as panel A but both ubi-PLSs and non-ubi-PLSs are stratified by association with Mendelian-disease genes.
D. Barplot shows the percentages of ubi-PLSs and non-ubi-PLSs that are associated with Mendelian-disease genes and cell-essential genes.
E. Same as panel A but both ubi-PLSs and non-ubi-PLSs are stratified by association with Mendelian-disease genes and cell-essential genes.
F. Word cloud of the top 100 Mendelian diseases most frequently associated with ubi-PLSs.
G. Word cloud of the top 100 Mendelian diseases most frequently associated with non-ubi-PLSs.
H. Scatter plot shows the percentage of genes implicated in a Mendelian disease whose TSSs overlap ubi-PLSs (x-axis) or non-ubi-PLSs (y-axis).
